# Comparative range-wide phylogeography and evolutionary history of the two *Pyrrhocorax* species (Passeriformes: Corvidae)

**DOI:** 10.7717/peerj.21799

**Published:** 2026-10-09

**Authors:** Iñigo Palacios-Martínez, Francisco Morinha, José Antonio Dávila, Mohammad Tohidifar, Michael Stubbe, Annegret Stubbe, Augusto De Sanctis, Stavros Xirouchakis, Guillermo Blanco

**Affiliations:** 1 Department of Evolutionary Ecology, Museo Nacional de Ciencias Naturales, CSIC, Consejo Superior de Investigaciones Científicas, Madrid, Spain; 2 Escuela de Doctorado UAM, Centro de Estudios de Posgrado, Universidad Autónoma de Madrid, Madrid, Spain; 3 Laboratory of Biodiversity and Molecular Genetics, Morinha Lab, Vila Real, Portugal; 4 Instituto de Investigación en Recursos Cinegéticos, IREC (CSIC, UCLM, JCCM), Consejo Superior de Investigaciones Científicas, Ciudad Real, Spain; 5 Iran Bird Records Committee, Tehran, Iran; 6 Martin-Luther Universität Halle-Wittenberg, Halle-Wittenberg, Germany; 7 Stazione Ornitologica Abruzzese, Pescara, Italy; 8 School of Sciences and Engineering, Natural History Museum of Crete, University Campus (Knossos), University of Crete, Crete, Greece

**Keywords:** Alpine chough, Conservation, Divergent mitochondrial clades, Introns, Mitochondrial DNA, Palearctic glaciations, Red-billed chough

## Abstract

Phylogeographic analyses provide key insights into how historical climate change, geographic barriers, and species-specific ecological traits shape evolutionary divergence. We investigated the phylogeography and evolutionary history of the two extant chough species, the red-billed chough (*Pyrrhocorax pyrrhocorax*, *n* = 65) and the alpine chough (*Pyrrhocorax graculus*, *n* = 33), across most of their Palearctic range. Using concatenated mitochondrial DNA sequences, complemented by nuclear intron markers, we reconstructed and test lineage divergence and historical dispersal patterns. Haplotype networks revealed long branches separating geographically isolated populations, indicating prolonged historical isolation. Phylogenetic analyses confirmed the monophyly of *Pyrrhocorax* and revealed a deep split between the two species at approximately 2.5 million years ago. Formal Ancestral Range Analysis (ARA) indicates that Himalayan regions served as the ancestral hub for both species. Within *P. graculus*, two main mitochondrial lineages were identified: a highly divergent Himalayan and European–Moroccan lineages (around 94 kya). In contrast, *P. pyrrhocorax* exhibited pronounced phylogeographic complexity, with major east–west differentiation and additional regional structuring across Europe, North Africa, Canary Islands, Ethiopia, and Central Asia, with divergence times ranging from  0.75 Mya to 61–94 kya. Quantitative metrics (*π* and *Da*) revealed significant interspecific divergence and distinct population-level structuring. Our results highlight the combined roles of historical isolation, Pleistocene climate dynamics, altitudinal gradients, and ecological constraints in shaping divergence in *Pyrrhocorax*. This phylogeographic framework provides essential guidance for conservation management, emphasizing the importance of preserving distinct evolutionary lineages in fragmented and declining populations.

## Introduction

Phylogeography and genetic structure in birds provide critical insights into evolutionary processes, including speciation, adaptation, and population divergence (Avise, 2000; Hewitt, 2001; Stewart et al., 2010). Modern approaches emphasize integrating comparative population genetics with geographic context to better resolve these histories (Edwards et al., 2022).Understanding population structure across space and time allows the inference of historical patterns of dispersal, connectivity, and demographic change (Lowe & Allendorf, 2010; Bradburd & Ralph, 2019), revealing how historical events, environmental variation, and life-history traits interact to shape genetic structure and diversity (Zink, 2004). Despite these advances, comprehensive studies across extensive geographic ranges (*i.e.,* Palearctic) remain scarce, constraining our understanding of the drivers of evolutionary differentiation in widely distributed species and their responses to environmental changes (Lira-Noriega & Manthey, 2014; Pironon et al., 2017). Such information is crucial for threatened species, as it can guide captive breeding, reintroduction or reinforcement programs by considering the genetic structure and origin of individuals, thereby maintaining genetic diversity and evolutionary potential.

Genetic differentiation in birds is influenced by a combination of extrinsic and intrinsic factors (Hartl & Clark, 2007; Orsini et al., 2013). Physical barriers such as mountain ranges, deserts, and large bodies of water can restrict movement and gene flow, promoting population divergence, while isolation by distance can further enhance differentiation between geographically distant populations (Slatkin, 1987; Newton, 2003). Historical climatic events, particularly Pleistocene glaciations, created periods of geographic isolation followed by postglacial recolonization, leaving lasting signatures on genetic diversity and population connectivity (Hewitt, 2001; Stewart et al., 2010). Intrinsic traits, including social behaviour, site fidelity, and ecological plasticity, modulate connectivity among populations, sometimes enabling gene flow even across apparently restrictive landscapes (Burg & Croxall, 2004; Saetre & Saether, 2010; Delmore & Irwin, 2014; Blanco et al., 2021a). Studying species with broad but fragmented distributions, including fully isolated breeding nuclei, provides valuable opportunities to explore how extrinsic and intrinsic factors interact over evolutionary timescales, revealing patterns applicable to other avian taxa (Lovette, 2004; Haring, Gamauf & Kryukov, 2007; Kryukov, 2019; Salinas et al., 2021).

While previous phylogeographic studies have highlighted the utility of widespread corvids as models for understanding evolutionary processes in the Palearctic (Kryukov et al.,, 2004; Kryukov, 2019, Kryukov, 2025; Haring, Gamauf & Kryukov, 2007; Poelstra et al., 2014; Vijay et al., 2016; Knief et al., 2019; Salinas et al., 2021), the genus *Pyrrhocorax* Tunstall, 1771 remains relatively underexplored (Wenzel et al., 2012; Morinha et al., 2016; Morinha et al., 2017). *Pyrrhocorax* represents one of the oldest and most distinct lineages within the Corvidae, forming a basal branch of the family (Ericson et al., 2005; Huang & Ruan, 2018). Its early divergence, Palearctic origin, and specialization to high-mountain habitats set it apart from other corvids (Ericson et al., 2005; Fernando, Peterson & Li, 2017). The two species of this genus, the red-billed chough (*P. pyrrhocorax*) and the alpine chough (*P. graculus*), offer a valuable system for examining how geographic barriers, past climate, and behavioural traits shape genetic diversity. Both species exhibit east–west Palearctic distributions with well-defined and often fragmented populations (Cramp & Perrins, 1994). This is particularly pronounced in *P. pyrrhocorax*, which occupies a wide range of cliff-associated habitats from the Iberian Peninsula to Central Asia, with several completely isolated populations, including those in La Palma (Canary Islands) and the Ethiopian highlands, which represent the southwestern and southeastern limits of its global distribution (Cramp & Perrins, 1994; Morinha et al., 2017).

*Pyrrhocorax* ecological breadth and population fragmentation provide a framework to assess how environmental variation and geographic isolation drive evolutionary divergence. Both species also have a rich fossil record, with remains frequently found in karstic deposits across their ranges, which provide historical context but are limited by dating precision and taxonomic resolution (Tyrberg, 1998; Tyrberg, 2008; Blanco, Sánchez-Marco & Negro, 2021b). Consequently, molecular analyses of contemporary populations are essential to reconstruct the evolutionary and biogeographic history of these species. Understanding this history is also critical for conservation, given the existence of multiple fragmented, declining, and extirpated populations (Morinha et al., 2017; Cross et al., 2023; Pennycott, Bignal & McCracken, 2025). This knowledge is particularly relevant for captive breeding, population reinforcement, and reintroduction programs, which depend on the accurate identification of evolutionary significant units (ESUs) and the preservation of local genetic adaptations (Attard et al., 2016; Willoughby et al., 2017). A robust phylogeographic framework can guide effective conservation across fragmented ranges by preventing genetic homogenization, ensuring the long-term viability of restored populations (Barbosa et al., 2018; Bobo-Pinilla et al., 2022).

The knowledge available from mitochondrial markers on the red-billed chough reveal relatively weak structure among populations from the Iberian Peninsula, Central Europe, and La Palma, with widely shared haplotypes and star-like networks reflecting a recent postglacial history and limited historical differentiation (Wenzel et al., 2012; Morinha et al., 2017; Morinha et al., 2020). However, populations from continental North Africa (Rif and Atlas Mountains in Morocco) constitute a clearly differentiated mitochondrial lineage (Morinha et al., 2020). In contrast, nuclear markers show extremely strong structuring, with multiple clearly differentiated genetic units, indicating recent restrictions to gene flow largely constrained by the species’ strict social structure and natal philopatry, which limit recruitment and mixing among populations despite the species’ high dispersal capacity (Morinha et al., 2017). Unfortunately, mitochondrial and nuclear population structure in *P. graculus* has not yet been investigated. Studying mitochondrial DNA across the species’ entire distribution is particularly valuable for revealing the evolutionary history shaped by past paleoclimatic events rather than by recent processes, which are more appropriately investigated using nuclear genomic methods, since these are expected to reveal complex structuring, as has been observed in populations from the Iberian Peninsula, and La Palma (Morinha et al., 2017; Recuerda et al., 2024).

In this study, we investigate the genetic structure and phylogeographic patterns of the genus *Pyrrhocorax* across most of their worldwide range. Our aim is to reconstruct the evolutionary history of *P. pyrrhocorax* and *P. graculus*, including the timing of their divergence, and to understand how past environmental changes and species-specific ecological and geoclimatic processes have shaped their genetic structure across their current distributions. To achieve this, we integrated phylogenetic and ancestral range analysis with a quantitative assessment of nucleotide diversity, and net divergence. We hypothesize that Pleistocene climatic oscillations and earlier geoclimatic episodes generated deep mitochondrial structuring, with expansions during postglacial and other geoclimatic periods contributing to the present genetic structure and distribution. Given their contrasting ecology and altitudinal ranges, we predict that *P. pyrrhocorax*, which occupies an exceptionally broad altitudinal range from sea level to high elevations in the Himalayas and a variety of habitats including steppes, semi-deserts, and coastal and mountainous areas, will exhibit more complex phylogeographic patterns than *P. graculus*, a high-mountain specialist largely restricted to alpine environments, which is expected to show more pronounced genetic clustering due to its ecological specialization. We further expect that historical refugia, geoclimatic episodes, and ecological differences have driven differential patterns of speciation, lineage diversification, and expansion. Ultimately, this study aims to provide a framework for understanding the interplay between biogeography, ecology, and evolutionary history, offering essential information for conservation, including the identification of evolutionary significant units to guide reintroduction and population reinforcement programs.

## Materials & Methods

### Study species

The two chough species studied are medium-sized, long-lived members of the crow family known for their strong social behaviour and high cognitive capacity. Both species exhibit high fidelity to their mates and nesting sites (Holyoak, 1972; Banda & Blanco, 2014). These birds primarily forage in open habitats and typically nest in natural cavities such as caves and rock crevices, as well as in artificial structures (Cramp & Perrins, 1994; Blanco et al., 1997).

Although they have broad Palearctic distributions across Europe, Africa, and Asia, they tend to be locally scarce (Cramp & Perrins, 1994). The geographic range of *P. pyrrhocorax* extends from western Ireland and Britain eastward across southern Europe, North Africa, and the mountainous regions of Anatolia, Central Asia, the Himalayas, Mongolia, and China. It inhabits coastal cliffs and high mountain areas, with isolated populations (BirdLife International, 2024). Likewise, *P. graculus* occupies high-altitude mountain systems across southern Europe, North Africa, and Asia, including the Pyrenees, Alps, Atlas, Caucasus, Balkans, Himalayas, and the Central Asian Highlands (BirdLife International, 2016).

Both *Pyrrhocorax* species are catalogued by the International Union for Conservation of Nature (IUCN) as Least Concern (LC), with stable populations along their distributions with local and regional declines, and extinctions (BirdLife International, 2016; BirdLife International, 2024). In addition, reintroduction and conservation programs has been designed for the *P. pyrrhocorax* in the British Isles (Corry et al., 2021; Consorte-McCrea, Franz & Bainbridge, 2023).

### Sampling and DNA extraction

Blood (*n* = 13 samples, *P. graculus n* = 3, *P. pyrrhocorax* = 10), and feathers (*n* = 85 samples, *P. graculus n* = 30, *P. pyrrhocorax n* = 55) were used for DNA extractions from the Iberian Peninsula (*P. graculus n* = 7, *P. pyrrhocorax* = 7), La Palma Island (*P. pyrrhocorax* = 9), United Kingdom (*P. pyrrhocorax* = 5), Italy (*P. graculus n* = 3, *P. pyrrhocorax* = 6), Austria (*P. graculus* = 6), Greece (*P. graculus n* = 5, *P. pyrrhocorax n* = 3), Bulgaria (*P. graculus n* = 2), Morocco (*P. graculus n* = 7, *P. pyrrhocorax n* = 8), Ethiopia (*P. pyrrhocorax n* = 4), Turkey (*P. pyrrhocorax n* = 2), Iran (*P. pyrrhocorax n* = 5), Mongolia (*P. pyrrhocorax n* = 11), Kazaksthan (*P. pyrrhocorax n* = 2), India (*P. graculus n* = 1), Nepal (*P. pyrrhocorax n* = 2) (Fig. 1A; Table S1). Molted feathers were collected in foraging areas, nesting sites, and communal roosts. Blood samples were collected by venipuncture of the sub-brachial vein, and stored in absolute ethanol. Genomic DNA was extracted using the Quick-DNA Miniprep Plus Kit (Zymo Research, Irvine, CA, USA) following the optimizations previously described for blood and feather samples (Morinha et al., 2017).

**Figure 1:**
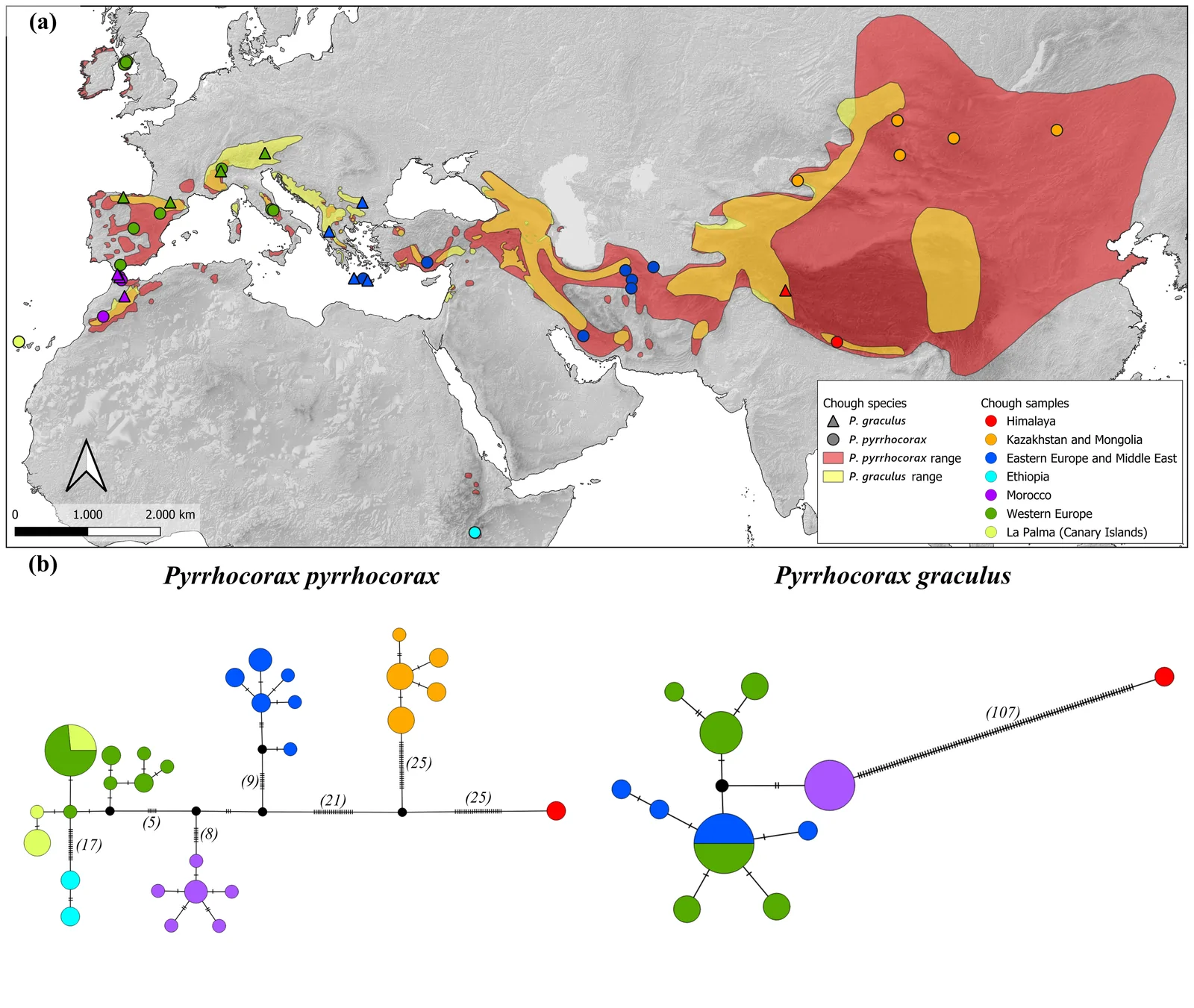
Distribution ranges and sampling scheme of both chough species (*Pyrrhocorax* Tunstall, 1771), and haplotype networks based on concatenated mtDNA data. (A) Distribution map of chough ranges. The yellow area indicates the known distribution range of *Pyrrhocorax graculus*, and the red area indicates the distribution range of *Pyrrhocorax pyrrhocorax* (BirdLife International, 2016; BirdLife International, 2024). Circular symbols denote sampling localities of *P. pyrrhocorax*, whereas triangular symbols denote sampling localities of *P. graculus*; colors correspond to different sampling regions as indicated in the legend. (B) Maximum-parsimony haplotype networks inferred from a concatenated mitochondrial dataset comprising a 512-bp fragment of *ND2*, a 638-bp fragment of the control region (*CR*), and a 644-bp fragment of *COI*, based on field samples of *P. pyrrhocorax* (left) and *P. graculus* (right). Circles represent haplotypes, with sizes proportional to haplotype frequency. Colors indicate geographic origin as shown in the legend in (A). Network branches correspond to single nucleotide substitutions, with additional mutations represented by bars along branches. Small black circles indicate unsampled or extinct haplotypes. Background topography is based on a 30 m resolution digital elevation model from NASA Earth Observatory (http://earthobservatory.nasa.gov/).

### Ethics statement

Field sampling of naturally molted feathers was conducted by the authors and collaborators during years when national regulations did not require specific permits for this type of material, as it did not involve capturing the birds or altering their normal behaviour. Samples from Greece were obtained by the authors from the historical collections housed in the Natural History Museum of Crete. Blood samples from Spain were obtained under official permits from the Spanish Ministry of the Environment and Regional Administrations, linked to capture, handling, and ringing permits from the Spanish Bird Ringing Centre (permit no. 530115 to G.B.). All procedures involving animal handling and tagging were conducted in strict accordance with the ethical standards and regulations established in Law 42/2007 on Natural Heritage and Biodiversity (Article 61, Ministerio para la Transición Ecológica y el Reto Demográfico de España), and in compliance with Spanish Royal Decree 53/2013 on the protection of animals used for experimentation and other scientific purposes (superseding previous regulations). This study also complies with the Animal Research: Reporting of *In Vivo* Experiments (ARRIVE) guidelines (https://arriveguidelines.org).

### Amplification and sequencing of mtDNA and nuclear markers

Polymerase chain reaction (PCR) was used to amplify three mitochondrial DNA fragments: the control region (*CR*) using primers JCR13 and H1248 (Saunders & Edwards, 2000), the nicotinamide adenine dinucleotide (NADH) dehydrogenase subunit 2 (*ND2*) with primers L5216 and H5766 (Sorenson et al., 1999; Sefc, Payne & Sorenson, 2003), and the cytochrome c oxidase subunit I (*COI*) with primers COIA and COIF (Palumbi et al., 1991); and the partial sequences of two nuclear introns: the transforming growth factor β 2 (*TGFB2*) with primers TGFB2.5F and TGFB2.6R (Primmer et al., 2002), and the Brahma gene (*BRM*) with primers BRM15F and BRM15R (Borge et al., 2005) (see Table 1).

**Table 1:** Primers used to amplify the partial mtDNA and nuclear genes and annealing temperatures (Ta) used in this study.

**Gene region**	**Primer sequences (5′–3′)**	**Ta (°C)**	**Amplicon length (bp)**	**Reference**
*CR*	JCR13: TGTTTTCTTTTTGGGGTCTCTTCAATAAGC H1248: CATCTTCAGTGTCATGCT	60	638	Saunders & Edwards (2000)
*ND2*	L5216: GGCCCATACCCCGRAAATG H5766: RGAKGAGAARGCYAGGATYTTKCG	58	512	Sorenson et al. (1999), Sefc, Payne & Sorenson (2003)
*COI*	COIA: AGTATAAGCGTCTGGGTAGTC COIF: CCTGCAGGAGGAGGAGAYCC	48	644	Palumbi et al. (1991)
*TGFB2*	TGFB2.5F: GAAGCGTGCTCTAGATGCTG TGFB2.6R: AGGCAGCAATTATCCTGCAC	65	590	Primmer et al. (2002)
*BRM*	BRM15F: AGCACCTTTGAACAGTGGTT BRM15R: TACTTTATGGAGACGACGGA	55	373	Borge et al. (2005)

PCR amplifications were carried out in a total volume of 20 µl. Each reaction included 10 µl of 2x MyTaq HS Mix (Bioline, Meridian Bioscience, Cincinnati, OH, USA), 5 pmol of each primer, ∼20 ng of template DNA and ultrapure DNase/RNase-free water to complete the total volume. The amplification protocol consisted of 95 °C for 5 min followed by 35 cycles of 95 °C for 30 s, annealing temperature according to the primers (Table 1) for 1 min, 72 °C for 30 s, and a final extension at 60 °C for 10 min. All gene regions successfully amplified were bi-directionally sequenced by Sanger sequencing. Sequences were visualized, edited and aligned using the Chromas software version 2.6.6 (https://technelysium.com.au/wp/chromas/) and Clustal Omega (Sievers et al., 2011). All sequences were deposited in GenBank (Table S1).

### Phylogenetic analyses

Nucleotide sequences were aligned using SEQUENCHER 4.1.4 (GeneCodes) and a final alignment was imported to DnaSP 6.11.01 (Rozas et al., 2017) to generate a haplotype data file for each marker. A median-joining network of mtDNA haplotypes was constructed using the program network PopART, version 1.7 (Leigh, Bryant & Nakagawa, 2015).

To estimate divergence times among major geographical lineages, we generated an ultrametric tree with BEAST v.2.6.6 (Bouckaert et al., 2014). Sequences of the outgroup species were extracted from the complete mitochondrial genomes available in GenBank for *Lanius collurio* (accession MN122863), *Daphoenositta chrysoptera* (accession KY994604), *Pachycephala pectoralis* (accession KY994600), and *Gymnorhina tibicen* (accession MK453379). Models of sequence evolution were estimated with jModelTest v.2.1.7 (Darriba, Taboada & Posada, 2012). The HKY+I+G model was used for *CR* and the HKY+G model were selected for *ND2* and *COI* (Hasegawa, Kishino & Yano, 1985; see values in Table 2). To estimate divergence times we applied a strict clock model to a topology-constrained tree using an average mutation rate of 0.029/site/Mya for *ND2* and 0.016/site/Mya for *COI* (Lerner et al., 2011), with a normal distribution for the rate prior, letting the program estimate the mutation rate for *CR*. The MCMC analysis was run for 100 million generations, sampling every 1,000 generations, with the first 10% discarded as burn-in. The convergence and mixing of the chains, and effective sample sizes (ESS) were analysed with Tracer v.1.7.1 (Rambaut et al., 2018). Bayesian posterior probabilities (BPP) were used to assess nodal support. The maximum clade credibility tree was visualized and edited with FIGTREE v. 1.4.4 (Rambaut, 2018).

**Table 2:** Evolutionary models and values used in phylogenetic inference according to Hasegawa, Kishino & Yano (1985).

**Region**	**Best fit model**	*α* ^a^	**I** ^b^
*CR*	HKY+I+G	0.940	0.232
*ND2*	HKY+G	0.129	–
*COI*	HKY+G	0.256	–

**Notes.**

a alpha shape parameter.

b proportion of invariable sites.

To test lineage boundaries and determine whether lineages represent distinct evolutionary units under the general lineage species concept, we performed Bayesian Species Delimitation (BSD) models using BPP v. 4.7 (Yang & Rannala, 2010; Yang, 2015). We utilized the reversible-jump Markov chain Monte Carlo (rjMCMC) algorithm to evaluate whether to collapse or retain nodes throughout the phylogeny based on the five-fragment concatenated dataset. An analysis A11 was employed, which explores species delimitation models and topologies by allowing the species tree to vary *via* nearest-neighbor interchange or sub-tree pruning and re-grafting (Yang & Rannala, 2014). We used algorithm 1 with values of 2 and 10 for the fine-tuning parameter to ensure that the rjMCMC mixed effectively in species delimitation models. We evaluated the impact of prior distributions on ancestral population size (θ) and divergence time (*τ*) by testing combinations of gamma priors (shape α = 2; scale β = 10, 100, 2000). Each analysis consisted of two independent chains run for 500,000 generations, with a burn-in of 50,000 and thinning every two generations.

### Ancestral area reconstruction analyses

Ancestral Range Analysis (ARA) for *Pyrrhocorax* was conducted using the *BioGeoBEARS* package in R (v.4.3) (Matzke, 2013). The calibrated phylogenetic tree was midpoint-rooted, pruned to include only relevant taxa, and resolved into a strictly bifurcating phylogeny using multi2di and collapse.singles functions, with zero or negative branch lengths adjusted to 0.001. We implemented the Dispersal-Extinction Cladogenesis (DEC) model (Ree & Smith, 2008) *via* maximum likelihood, with parameters optimized using the *optimx* package. Based on *Pyrrhocorax* distributions, seven biogeographic areas were defined and the maximum range size was restricted to three areas to prevent biologically improbable state combinations. Ancestral states were inferred and plotted onto the phylogeny to visualize range evolution.

### Population structure and diversity

To estimate genetic divergence between populations, we calculated net nucleotide divergence (*Da*) using the formula *Da* = *Dxy*–(*Dx* + *Dy*)/2, where *Dxy* represents the average pairwise divergence between populations, and *Dx* and *Dy* represent the average pairwise differences within populations (nucleotide diversity, *π*). To characterize the genetic diversity within each population, we calculated *π*, and to examine the relationship between genetic distance and population divergence, we constructed a matrix comparing pairwise *Da* values across all population pairs. These calculations were performed using DnaSP. For all analyses involving mitochondrial data, the loci were treated as a single concatenated mtDNA alignment to account for the linked nature of the mitochondrial genome.

## Results

Partial sequences of the mitochondrial genes *COI* (644 bp), *ND2* (512 bp), and *CR* (638 bp), as well as the nuclear introns *TGFB2* (590 bp) and *BRM* (373 bp), were successfully obtained for 98 *Pyrrhocorax* specimens (Table 1), except for four specimens lacking sequences for the *TGFB2* intron (Table S1). A greater number of haplotypes was detected in the mitochondrial markers (*COI*: *P. pyrrhocorax n* = 16, *P. graculus n* = 4; *ND2*: *P. pyrrhocorax n* = 12, *P. graculus n* = 7; CR: *P. pyrrhocorax n* = 18, *P. graculus n* = 4) than in the nuclear introns (*TGFB2*: *P. pyrrhocorax n* = 5, *P. graculus n* = 2; *BRM*: *P. pyrrhocorax n* = 4, *P. graculus n* = 5) (Fig. 2). Consistent with this pattern, mitochondrial haplotype networks exhibited greater numbers of substitutions between the two chough species than did the intron networks (Fig. 2).

**Figure 2:**
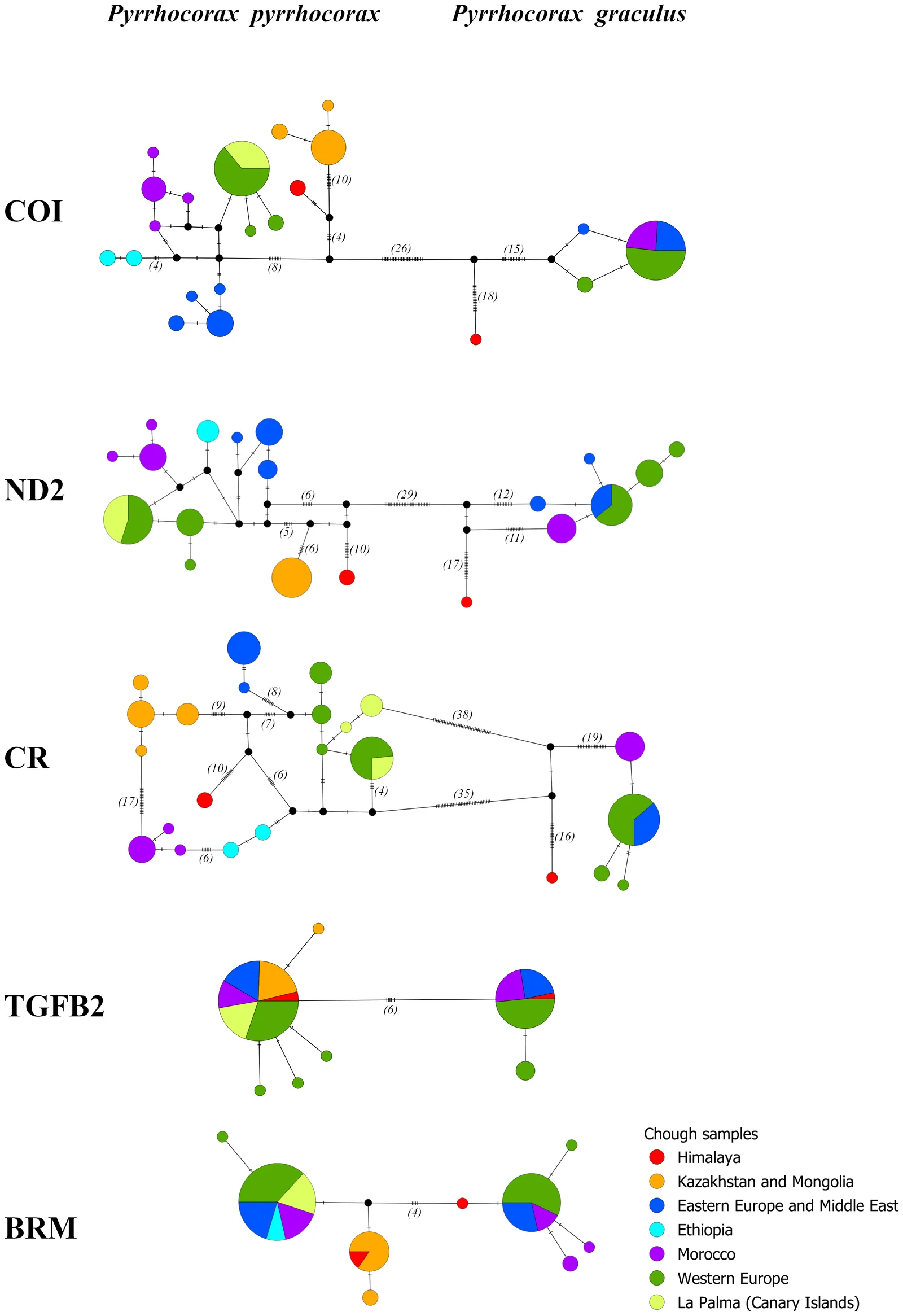
Haplotype networks. Haplotype networks of 644 bp of the *COI* fragment, 512 bp fragment of the *ND2* gene, 638 bp fragment of the *CR*, 590 bp fragment of *TGFB2*, and 373 bp fragment *BRM* from field samples of alpine chough (*Pyrrhocorax graculus*) and red-billed chough (*Pyrrhocorax pyrrhocorax*). Circles correspond to haplotypes, and their size is proportional to the haplotype’s frequency. Network branches represent a single nucleotide change, with additional mutations indicated by bars along branches. Branches with three or more mutations are labelled for better interpretation. Small black circles represent unsampled or extinct haplotypes.

Haplotype networks based on the mitochondrial markers COI, CR, and ND2 revealed two well-defined haplogroups in *P. graculus*, whereas *P. pyrrhocorax* was structured into six clearly differentiated haplogroups (Figs. 1B, 2). In *P. graculus*, Himalayan haplotypes were strongly isolated from all others, differing by 107 substitutions from the nearest haplotype. A comparable pattern was observed in *P. pyrrhocorax*, where Himalayan haplotypes were separated from the closest lineage by 51 substitutions (Fig. 1B). In *P. pyrrhocorax*, Moroccan haplotypes were separated from European haplotypes by 17 substitutions, indicating a higher level of genetic differentiation than in *P. graculus*, where Moroccan and European haplotypes differed by only four substitutions (Fig. 1B). A similar contrast was found between western and eastern European populations: these groups were intermixed in *P. graculus*, whereas in *P. pyrrhocorax* they were separated by 24 substitutions (Fig. 1B).

Although the overall geographic cohesion of haplotypes was consistent across mitochondrial markers, network topology varied slightly among loci, showing minor differences in the number of substitutions separating haplotype cores as well as subtle interspecific contrasts (*e.g.*, *COI*: 53; *ND2*: 52; *CR*: 57 substitutions; Fig. 2). Intron haplotype networks were generally more conserved than mitochondrial networks but still reflected the main phylogeographic structure of both taxa in the *BRM* locus. In contrast, the *TGFB2* locus did not recover this structure, grouping Himalayan choughs with the remaining haplotypes of their respective species (Fig. 2).

Bayesian ultrametric tree of concatenated mtDNA fragments (*ND2*, *CR*, and *COI*) from sampled individuals (Table S1) revealed a well-supported tree topology at the basal nodes (Table 3; Fig. 3). The genus *Pyrrhocorax* is strongly supported as monophyletic (BPP = 0.97), comprising two distinct lineages corresponding to the two extant species, (BPP = 1.0). Divergence between *Pyrrhocorax* and other taxa is estimated at 5.4 Mya (95% highest posterior density (HPD): 4.2–6.7 Mya), with the split between the two chough species occurring around 2.5 Mya (95% HPD: 2.0–3.1 Mya) (Table 3; Fig. 3).

**Table 3:** Summary of divergence estimates between major evolutionary splits in *Pyrrhocorax*. Split number corresponds to the order of inferred divergence events in Fig. 2. Node split indicates the clade separation of the lineages involved, while mean node age and its 95% confidence interval (in million years, Mya) were inferred from Bayesian phylogenetic analysis. BPP refers to Bayesian Posterior Probabilities for each node.

**Split**	**Node split**	**Node age mean (Mya)**	**95% Confidence interval (Mya)**	**BPP**
1	Divergence of *Pyrrhocorax*	5.403	[4.202-6.691]	0.97
2	*P. graculus*–*P. pyrrhocorax*	2.521	[1.993-3.080]	1.00
3	*P. graculus* Clade JKL–M	1.223	[0.926-1.550]	1.00
4	*P. pyrrhocorax* Clade ABCDEF–GH	0.750	[0.569-0.944]	1.00
5	*P. pyrrhocorax* Clade G–H	0.565	[0.400-0.732]	1.00
6	*P. pyrrhocorax* Clade ABCDE–F	0.327	[0.229-0.429]	1.00
7	*P. pyrrhocorax* Clade ABCD–E	0.258	[0.178-0.349]	0.95
8	*P. pyrrhocorax* Clade ABC–D	0.207	[0.138-0.282]	0.98
9	*P. graculus* Clade JK–L	0.094	[0.053-0.140]	1.00
10	*P. pyrrhocorax* Clade AB–C	0.086	[0.049-0.126]	1.00
11	*P. pyrrhocorax* Clade G1–G2	0.080	[0.015-0.188]	1.00
12	*P. graculus* Clade J–K	0.072	[0.023-0.208]	0.81
13	*P. pyrrhocorax* Clade A–B	0.061	[0.032-0.092]	0.98

**Figure 3:**
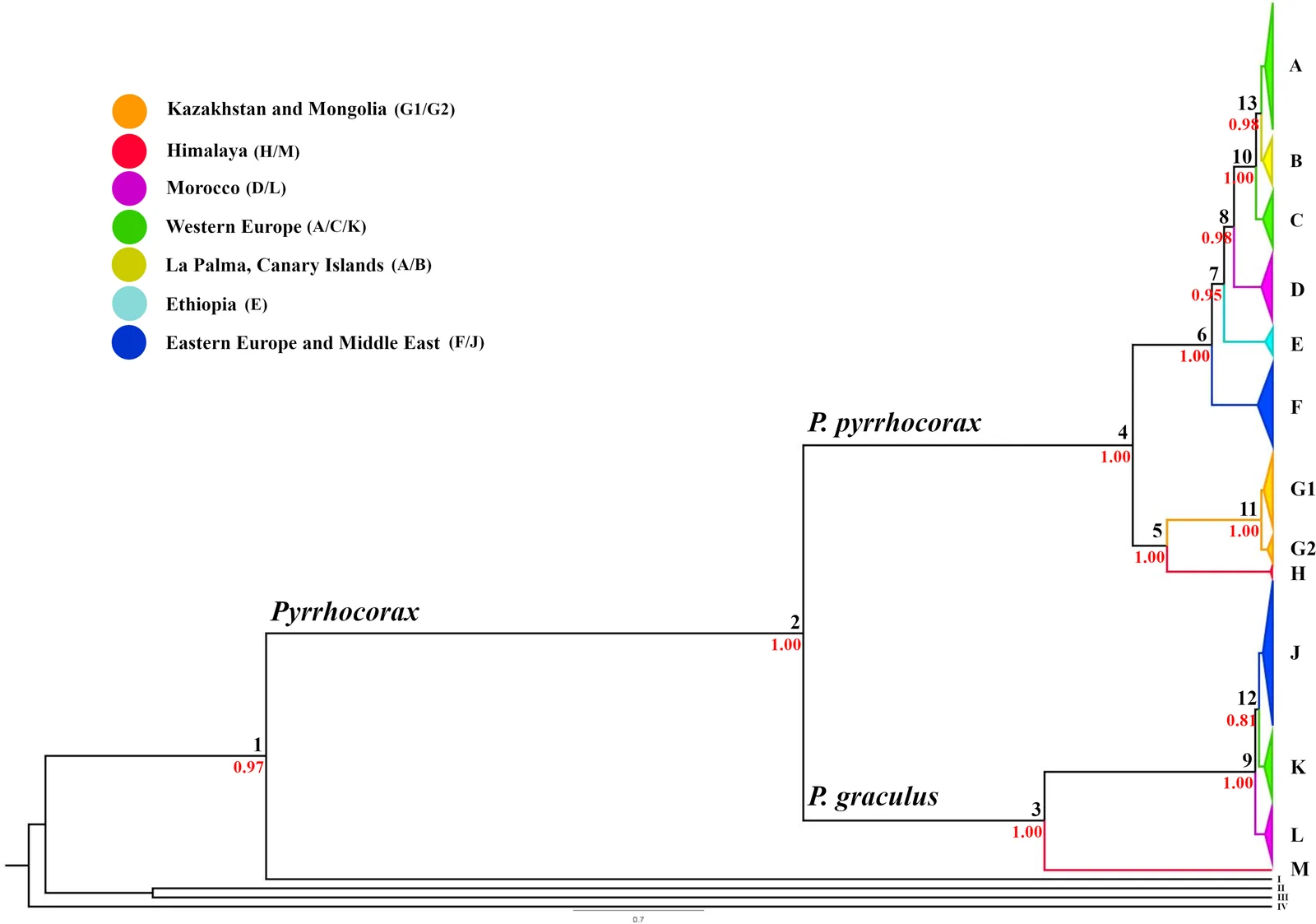
Bayesian inference phylogeny of *Pyrrhocorax* based on the combined mtDNA data set of *ND2*, *CR*, and *COI*, fragments. Colour-coded branches represent the different lineages of both chough species *P. pyrrhocorax*, and *P. graculus*. Genetic clades are signalized at the right side of the phylogeny (A–H for *P. pyrrhocorax*; and J–M for *P. graculus*). Numbers in black at branches correspond to the main splits within *Pyrrhocorax* phylogeny corresponding to split number in Table 3, and numbers in red represent the Bayesian Posterior Probability (BPP) values of each node split. Outgroups are labelled: *Lanius collurio* (I); *Daphoenositta chrysoptera* (II); *Pachycephala pectoralis* (III); and *Gymnorhina tibicen* (IV).

In *P. pyrrhocorax*, two main clades are also recovered. The eastern clade (comprising Himalayan -Clade H-, Mongolian, and Kazakh individuals -Clade G-) diverged from western populations about 0.75 Mya (95% HPD: 0.57–0.94 Mya; BPP = 1.0). Within this group, Himalayan choughs separated from Kazakh and Mongolian populations around 0.56 Mya (95% HPD: 0.40–0.73 Mya), followed by a divergence of Mongolian choughs (Clade G1-G2) around 80 kya (95% HPD: 15–188 kya; BPP = 1.0). Central Asian and Eastern European choughs (Clade F) split from western clades approximately 0.33 Mya (95% HPD: 0.23–0.43 Mya), while Ethiopian choughs (Clade E) diverged from other western populations around 0.26 Mya (95% HPD: 0.18–0.35 Mya; BPP = 0.95). Moroccan choughs (Clade D) diverged from continental European populations around 94 kya (95% HPD: 53–140 kya; BPP = 1.0), with further divergence among European populations (Clade AB-C) estimated at 86 kya (95% HPD: 49–126 kya; BPP = 1.0). A subsequent split between choughs from the British Isles, Italy, and Iberian Peninsula (Clade A) and those from La Palma (Clade B) occurred approximately 61 kya (95% HPD: 32–92 kya; BPP = 0.98; Table 3, Fig. 3). Consequently, sequences from Italy and Iberia are represented across both clades A and C, whereas sequences from the British Isles are only clustered in Clade A. Finally, some sequences from La Palma also fall within Clade A.

Within the *P. graculus* clade, two major subclades are identified (BPP = 1.0). The Himalayan specimen (Clade M) forms a distinct lineage that diverged approximately 1.2 Mya (95% HPD: 0.9–1.5 Mya). The sister subclade (Clade JKL) includes European (Clade JK) and Moroccan individuals (Clade L), with European populations diverging from Moroccan ones around 94 kya (95% HPD: 53–140 kya; BPP = 1.0). Subsequent divergence between Eastern (Clade J) and Western (Clade K) European groups occurred approximately 72 kya (95% HPD: 23–208 kya), though with lower support (BPP = 0.81; Table 3, Fig. 3).

The BSD model based on the estimated species tree strongly supported the a priori hypothesis of two well-differentiated species, with a posterior probability of 1.00 for this bipartition under the A11 algorithm. Within this framework, the full model of genetically distinct lineages (nine within *P. pyrrhocorax* and four within *P. graculus*) received varying support depending on prior configurations and Markov chain Monte Carlo (MCMC) tunings. The prior combination of β = 2,000 received strong support (posterior probability (PP) = 1.00) for the 13-lineage assumption across all runs. However, reducing the informativeness of the priors (β = 100 and β = 10) produced a progressive decrease in support for the full-lineage model. Specifically, with β = 100, support dropped to PP = 0.92 (with a PP = 0.07 probability of G1-G2 clade collapse), while runs with β = 10 showed further reductions down to PP = 0.00. Under β = 10 and a fine-tuning parameter of 2, the *P. pyrrhocorax* G clade entirely collapsed (PP = 1.00 for a 12-lineage hypothesis; Fig. 4). With a fine-tuning parameter of 10, the model collapsed to a 9-lineage delimitation, where *P. graculus* J, K, and L clades collapsed (PP = 1.00), and the *P. pyrrhocorax* G clade (PP = 1.00) and H clade (PP = 0.71) also collapsed (Fig. 4).

**Figure 4:**
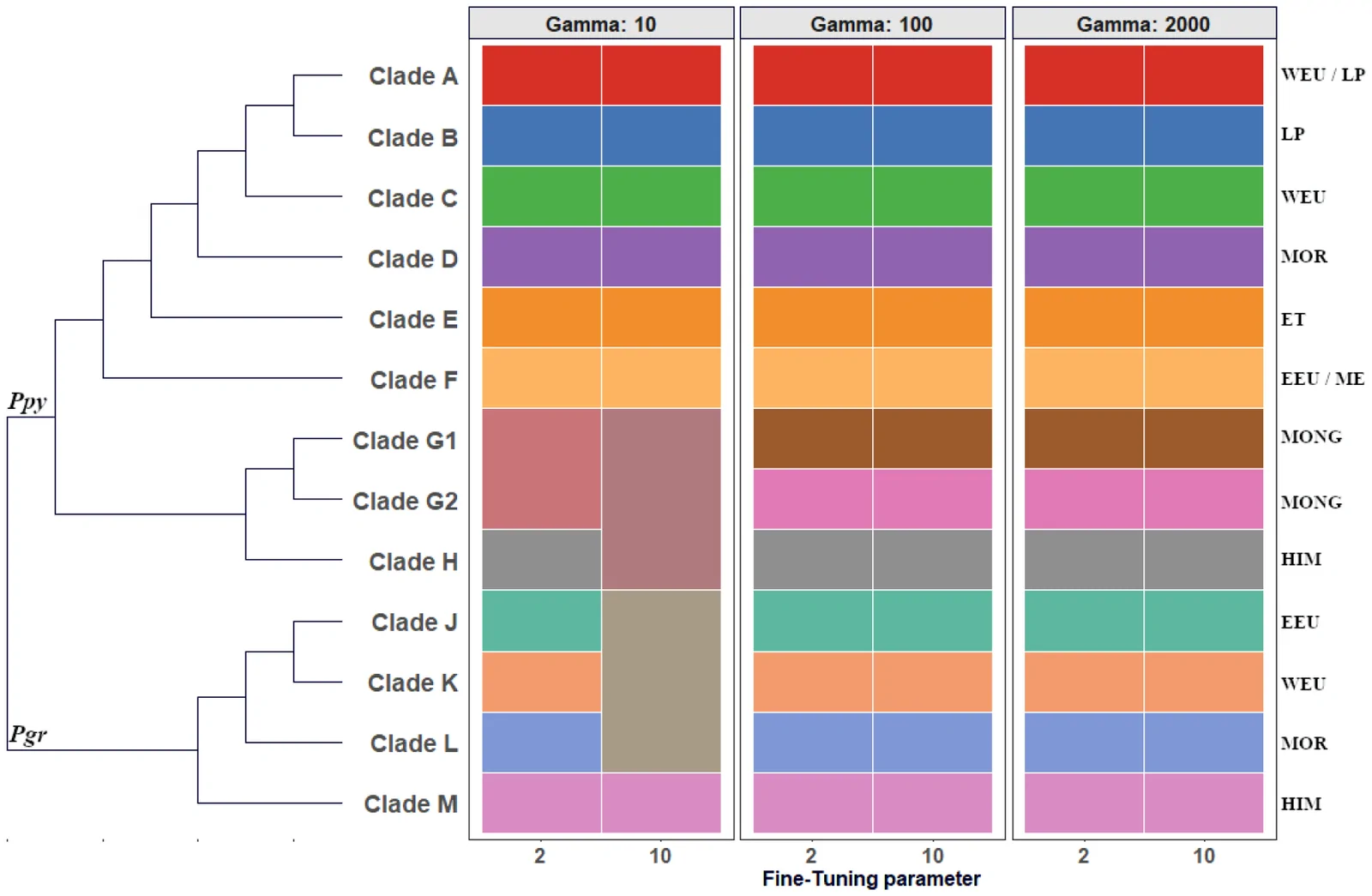
Bayesian species delimitation (BSD) performance across different prior configurations and fine-tuning parameters. The left panel shows the guide species tree used for the analyses, including the 13 a priori lineages (nine within *Pyrrhocorax pyrrhocorax –Ppy–* and four within *P. graculus –Pgr–*). Genetic clades characterization is referred to Fig. 3 and Table 3. The right panels summarize lineage delimitation outcomes under three β prior settings (10, 100, and 2000) and two fine-tuning values (2 and 10). Colored blocks indicate lineages inferred as genetically distinct, whereas merged blocks represent collapsed lineages. The geographic distribution of each clade is indicated on the right: Himalayas (HIM); Mongolia and Kazakhstan (MONG); ME (Middle East); Ethiopia (ET); Morocco (MOR); EEU (eastern Europe); WEU (western Europe); La Palma, Canary Islands (LP).

ARA indicates distinct geographic origins for *Pyrrhocorax* lineages. For *P. pyrrhocorax*, the common ancestor of the eastern (GH) clades likely originated in the eastern Palearctic, specifically within the Himalayan region (Region F). Conversely, the common ancestor of the western lineages is inferred to have occupied the Middle East (Region A). Within the ABCDE lineages, the ancestral range is localized to North Africa (Ethiopia and Morocco; Region BG), whereas the ancestor of the European-Moroccan clade originated in the western Mediterranean (Region CG; Fig. 5; Table S2). Furthermore, the ancestral lineage of the La Palma population is traced to western Europe (Region C). For *P. graculus*, the common ancestor of the western (JKL) clades was inferred to inhabit a broad range primarily spanning the Middle East (Region A), while ancestral states for the more basal nodes were most likely associated with the Himalayan region (Region F; Fig. 5; Table S2).

**Figure 5:**
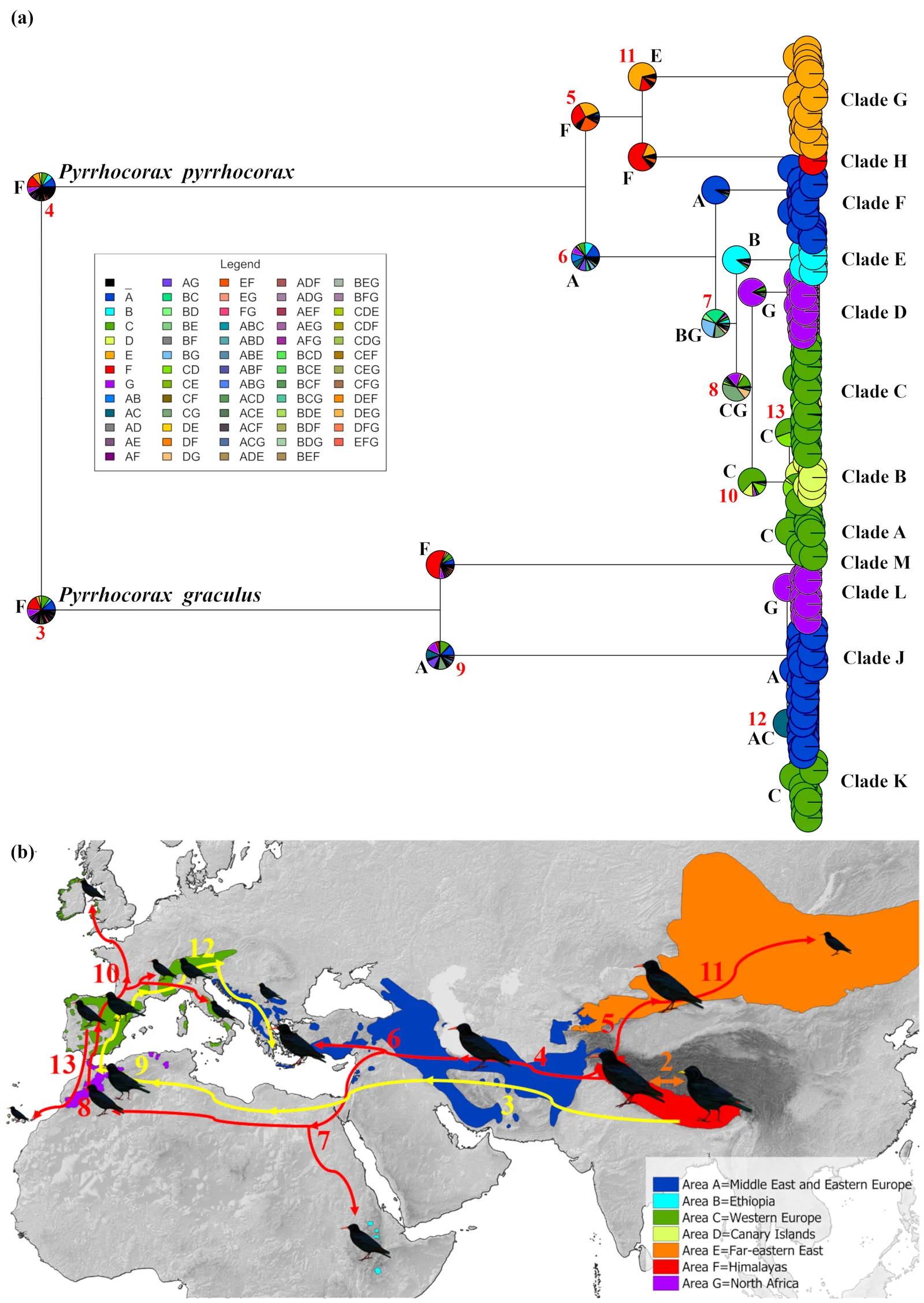
Ancestral area reconstruction for *Pyrrhocorax*. (A) Pie charts at internal tree nodes represent the relative probabilities of ancestral range combinations, inferred using *BioGeoBEARS* package. The most probable ancestor is indicated in capital letters in each main node. Maximum-likelihood marginal probabilities (%) for each ancestral geographic state at the major nodes are provided in Table S2. Combined ancestral ranges are indicated by the color-coded legend. The characterization of the genetic clades and red numbers indicating divergence splits are provided in Fig. 3 and Table 3. (B) Schematic model of the hypothesized evolutionary history of *Pyrrhocorax*, highlighting major divergence events and estimated times of lineage splits inferred from the mitochondrial phylogeny (Fig. 3) calibrated using relaxed molecular clocks. The orange arrow represents the separation of both species. Red and yellow arrows indicate the hypothesized dispersal routes across Eurasia and North Africa for *P. pyrrhocorax* and *P. graculus*, respectively. Chough icons represent sampled areas; the size of these icons decreases according to the estimated divergence age of the different clades. Numbers indicate the split number as reported in Fig. 3 and Table 3. Background topography is based on a 30 m resolution digital elevation model from NASA Earth Observatory (http://earthobservatory.nasa.gov/).

Nucleotide diversity (*π*) varied significantly across the eleven geographic populations, with higher diversity observed in *P. pyrrhocorax* (mean *π* = 0.01744) compared to *P. graculus* (mean *π* = 0.0049; Fig. 6). The highest diversity was found in western European *P. pyrrhocorax* (*π* = 0.00130) and Middle East *P. pyrrhocorax* (*π* = 0.00116), while both species in the Himalayan region, and *P. graculus* in Morocco exhibited the lowest levels of genetic diversity (*π* = 0.0). Net *Da* confirmed deep evolutionary separation between the two species, with pairwise *Da* values exceeding 0.09 in inter-specific comparisons. In contrast, intra-specific comparisons within *P. graculus* revealed lower *Da* values (0.0004 to 0.0366), indicating more recent divergence or ongoing gene flow (Fig. 6).

**Figure 6:**
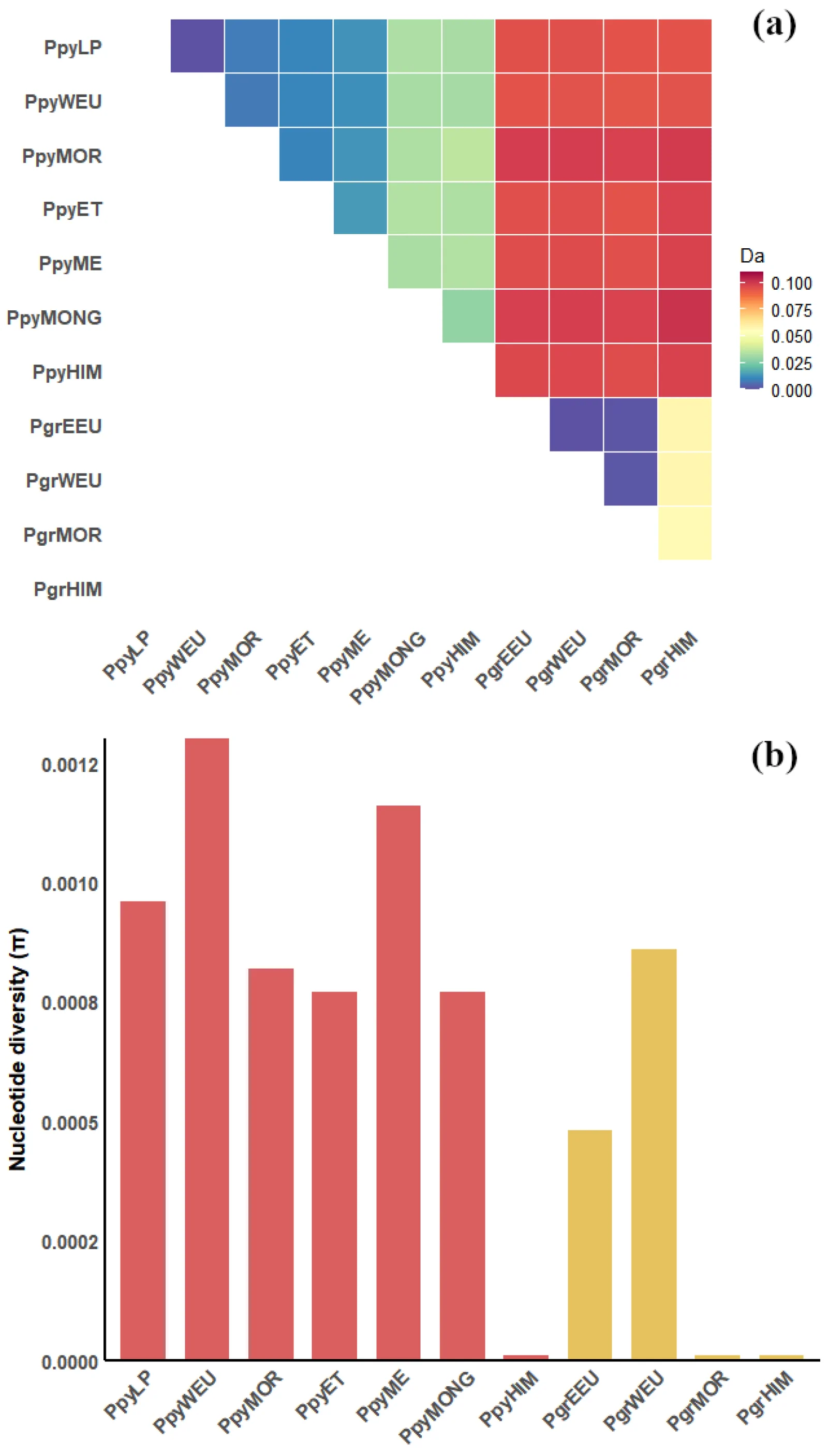
Genetic diversity and population divergence of *Pyrrhocorax*. (A) Matrix of net nucleotide divergence (*Da*) between identified geographic populations. Values above the diagonal represent pairwise net between-lineage divergence, visualized using a spectral color palette to highlight the magnitude of differentiation. (B) Barplot illustrating nucleotide diversity (*π*) across the studied populations. Species are color-coded: *P. pyrrhocorax* –Ppy–populations are shown in red and *P. graculus*–Pgr–in yellow. Population codes are as follows: *P. pyrrhocorax* (PpyLP: La Palma, Canary Islands; PpyWEU: western Europe; PpyMOR: Morocco; PpyET: Ethiopia; PpyME: Middle East and eastern Europe; PpyMONG: Far Eastern Asia (Mongolia and Kazakhstan); PpyHIM: Himalayas) and *P. graculus* (PgrEEU: eastern Europe; PgrWEU: western Europe; PgrMOR: Morocco; PgrHIM: Himalayas).

## Discussion

This study provides a comprehensive overview of the phylogeography of both species of *Pyrrhocorax* choughs across most of their respective, often shared, worldwide ranges. Within *P. pyrrhocorax*, we identified clear differentiation between eastern and western populations, with further structuring among western European, La Palma, North African, Middle East and eastern European, Ethiopian, and eastern Asian groups, while *P. graculus* shows a major separation between Himalayan and European–Moroccan lineages. Haplotype networks reveal long branches separating geographically isolated populations, and nuclear intron markers, although more conserved, largely support the same structure. BSD analyses robustly resolved boundaries across nearly all identified lineages. Furthermore, both species likely exhibit similar colonization histories and range expansion dynamics, following comparable dispersal routes, except in the case of the Eastern European enclaves, where asymmetric patterns of dispersal are evidenced.

*P. pyrrhocorax* may be expected to show less genetic structuring and more population homogeneity given its wider ecological niche in comparison to its congener. However, *P. pyrrhocorax* strong phylogeographic differentiation indicates that the potential homogenizing effect of its ecological plasticity has been outweighed by dispersal limitation and population cluster isolation. Consistent with these findings, our analysis of genetic diversity and divergence corroborates deep phylogeographic structuring: *P. pyrrhocorax* exhibits significantly higher nucleotide diversity than *P. graculus*, suggesting more stable historical population sizes or more extensive or recent range connectivity. The *Da* values further reinforces the deep evolutionary break between these two species highlighting how the broader ecological plasticity of *P. pyrrhocorax*, which is not restricted to alpine habitats, has enabled greater historical dispersal. These finding suggest that, since the group’s origin in the Himalayan–Tibetan region, long-term historical isolation and restricted modern gene flow have been the main drivers of divergence, likely driven by significant paleoclimatic events that shaped environmental barriers and promoted ecological isolation

### Evolutionary history of *Pyrrhocorax*

The evolutionary origins of the Corvidae family are hypothesized to lie within the East Palearctic—specifically the East Asian and Indo-Malayan regions (Fernando, Peterson & Li, 2017)—or potentially in Australia (Sibley & Ahlquist, 1985; Ericson et al., 2005), followed by potential westward dispersal. Drawing on the phylogenetic framework of Jønsson et al. (2016), which places *Pyrrhocorax* on a basal long branch within the Corvidae, and although our ARA analysis yielded low support regarding the precise origin of the genus, the most basal lineages for both *P. pyrrhocorax* and *P. graculus* are concentrated within the Himalayas, pointing to this region as the most plausible origin of both species.

Against this backdrop, the south-eastern Qinghai-Tibet Plateau (QTP) served as an “evolutionary cradle” and hub of ongoing diversification for plants, birds, mammals, and beetles (Deng et al., 2011; Schmidt et al., 2012; Zhang et al., 2014; Pisano et al., 2015; Ren, Conti & Salamin, 2015). In addition, several studies have shown that many passerine species (Passeriformes) experienced substantial Miocene divergence between shrubland-associated lineages and those adapted to high alpine environments (Drovetski et al., 2013; Tietze et al., 2013). Following a slowdown in morphological and physiological evolution due to niche saturation, early splits generated pronounced differences in body size and trophic strategies across clades (Kennedy et al., 2012; Qu et al., 2013; Price et al., 2014).

Although the timing and magnitude of QTP uplift remain subjects of intense debate, with geological evidence suggesting that significant portions of the plateau reached high elevations as early as the mid-Miocene (13–15 Mya), the subsequent diversification of *Pyrrhocorax* lineages appears consistent with the prolonged landscape and climatic transformations that characterized the Pliocene and Pleistocene (Renner, 2016). The divergence between *P. pyrrhocorax* and *P. graculus* aligns with these late-stage environmental shifts, supporting a key role for elevation gradients in driving their speciation, consistent with patterns observed in other avian taxa (Ding, Liao & Liu, 2020; Päckert et al., 2020a). Within this macroevolutionary context, the basal phylogenetic position of *Pyrrhocorax* isolated in a long branch within an earliest-diverging clade within Corvidae (Jønsson et al., 2016), provides a unique perspective on the early ecological evolution of the family. Although *Pyrrhocorax* itself is not forest-dwelling, the common ancestor of corvids is intuitively inferred to be likely associated with forested environments (Ericson et al., 2005). This interpretation is further supported by the ecology of the remaining forest-specialized genera such as *Dendrocitta*, *Crypsirina*, *Platysmurus*, and *Temnurus*, which are the closest phylogenetic relatives of *Pyrrhocorax* (Ericson et al., 2005; Jønsson et al., 2016; Fernando, Peterson & Li, 2017; Garcia-Porta et al., 2022). Against this evolutionary background, the lineage leading to *Pyrrhocorax* likely underwent an early and profound ecological transition, accompanied by anatomical specialization along steep environmental gradients (Laiolo & Rolando, 2001; Laiolo et al., 2004), potentially driven by peripatric or parapatric divergence. This transition probably involved a shift from ancestral montane forests of ancestor species to open alpine habitats of *Pyrrhocorax*, followed by the subsequent expansion of *P. pyrrhocorax* into arid and semi-desert environments. Despite this long evolutionary history in open habitats, both *Pyrrhocorax* species retain remarkable ecological flexibility. Such versatility may reflect the retention of ancestral ecological plasticity and, in some cases, the use of habitats resembling the forested environments inferred for the early corvid ancestor. For example, the isolated population of *P. pyrrhocorax* on La Palma forages within forested habitats (Blanco, Laiolo & Fargallo, 2014) and nests in endemic Canary Island palms (*Phoenix canariensis*) and Canary Island pines (*Pinus canariensis*) alongside their most common cliff nests (Blanco et al., 2026). Similarly, *P. graculus* is not exclusively confined to alpine environments, locally occupying low-elevation coastal mountains and marine cliffs for nesting, such as along the Rif coast of Morocco and in the Cantabrian Mountains of northern Spain ((SEO BirdLife, 2022); authors’ personal observations). Together, these phylogenetic and ecological lines of evidence reinforce the hypothesis that the deepest ancestral roots of the genus *Pyrrhocorax* were intimately tied to forested habitats before their subsequent radiation into open biomes.

Intraspecific divergence within *Pyrrhocorax* reflects patterns described in other alpine species, driven by altitudinal and latitudinal shifts throughout the Pleistocene (Lei, Qu & Song, 2014; Päckert et al., 2020b). However, the genus’ phylogeographic complexity transcends simple peripheral refugia models (Provan & Bennett, 2008; Rota et al., 2024). Instead, our results support a bidirectional exchange paradigm, where the QTP and its margins functioned as a dynamic, interconnected system rather than a source of unidirectional expansion (Song et al., 2018; Päckert et al., 2020b). This genetic architecture reflects an interplay between ancient Pliocene vicariance, driven by QTP aridification (Nie et al., 2017), habitat fragmentation (Gao et al., 2015), and recent cyclical dispersal (Yang, Dong & Lei, 2009). While Pleistocene glaciations influenced population structure, complex refugial dynamics fostered high-altitude stability, contrasting with the volatile demographic expansions seen in lowland taxa (Qu et al., 2010; An et al., 2020). Additionally, intermittent glacial corridors facilitated periodic admixture, tempering allopatric divergence (Qu et al., 2011; Ravinet et al., 2018; Päckert et al., 2019).

These dynamics underscore the complex interplay of demographic history, ecological specialization and macroevolutionary processes within Corvoidea (Jønsson et al., 2016; McCullough et al., 2023). A mixed model involving peripatric speciation, ecological parapatry, and secondary sympatry best accounts for the observed patterns, paralleling diversification in the *Carpodacus pulcherrimus* complex (Tietze et al., 2013; Päckert et al., 2020b). Choughs, unlike forest-dependent taxa, likely benefited from increasing aridity and the retreat of mesophilic forests in the QTP, which may have facilitated establishment and range expansions. During the Pleistocene, both species likely underwent southward dispersal toward the southeastern QTP in a process described as the “Himalayan exile” (Martens, 2015). Subsequent intensification of glacial cycles during the Mid-Pleistocene Transition, approximately 1.25–0.7 Mya (Päckert et al., 2020b), may have promoted westward expansion of *P. graculus* into the western Himalayas and potentially the Caucasus. The strong flight capabilities and adaptation to high-altitude and open habitats likely facilitated these movements, whereas subsequent isolation may have limited gene flow and reinforced divergence (Morinha et al., 2017). In this regard, the phylogeographic patterns of choughs provide a robust basis for modeling the distribution, colonization, and speciation of other open-land and montane birds with broad altitudinal ranges.

### Interspecific differences in phylogeography

Our ARA analysis suggest that *P. pyrrhocorax* likely began its differentiation in the QTP before spreading *via* two primary peripatric events. A southwestern lineage would have expanded across the Elburz, Hindukush, and western Himalayas, becoming isolated around Marine Isotope Stage (MIS) 18, approximately 0.75 Mya, during the Naynayxungla Glaciation (Zheng, Xu & Shen, 2002), while a second, northerly lineage would have diversified in Central Asia and Mongolia (∼0.56 Mya) as aridification increased (Guo et al., 2009; Hao et al., 2015). High regional haplotype diversity underscores these distinct evolutionary trajectories, which are further supported by the variation in *π* and *Da* observed across these clusters. Although BSD did not fully resolve the separation of lineages within the far-eastern and Himalayas, suggesting that these clusters may have remained connected during historical glacial oscillations, this incomplete resolution is directly reflected in the lower tree support observed for the far-eastern splits. Further westward, arid phases in the Middle Pleistocene would have facilitated dispersal through the Levantine corridor. By MIS 9 (∼330–300 kya), one of the warmest and most stable interglacials of the Pleistocene (Zazo et al., 2003; Green et al., 2006), populations likely established in the North of African continent, since the Nile Valley to the Atlas Mountains, potentially continuing into the Rift Valley. Repeated subsequent humid–arid cycles likely drove further fragmentation and regional isolation during MIS 7 (∼245–190 kya) in this area (Castañeda et al., 2009; Zachos & Habel, 2011). By MIS 6 (∼200 kya), intensified aridification would have created ecological barriers in North Africa (Leroy & Dupont, 1994; Touchan et al., 2011), potentially reducing gene flow between North African and Iberian populations despite their geographic proximity (Morinha et al., 2020), and promoting the establishment of populations in the Iberian Peninsula and other Mediterranean refugia. Posterior climatic improvement may have enabled colonization of northern regions in Europe. The western Iberian clade may have persisted in both inland and coastal refugia, and mitochondrial evidence suggests that populations from the Canary Islands likely derive from these currently extinct coastal populations, which were probably connected to Iberian populations through Moroccan coast during the Late Pleistocene, since 60 kya, with probably continuous colonization events (Morinha et al., 2020).

The phylogeographic history of *P. graculus* appears more constrained, likely due to its alpine habitat preference. Notably, despite its pronounced mitochondrial divergence, the Himalayan individual clustered with western populations at the *TGFB2* nuclear locus, failing to support a plausible hypothesis of species-level differentiation. Its preference for alpine habitats probably constrained its expansion capacity into more arid or lowland regions. Furthermore, the BSD analysis collapsed these western lineages, suggesting continuous or more recent interflux among them under optimal paleoclimatic conditions. *P. graculus* likely began its westward spread ∼1.2 Mya, potentially reaching the Atlas Mountains and the Iberian Peninsula (∼90 kya) through colonization pulses distinct from its congener. In areas of sympatry, such as the Balkans and Crete, the distinct inferred colonization routes of these two species (*P. pyrrhocorax* likely from the east, and *P. graculus via* a western route through North Africa and Iberia) represent a significant biogeographic difference that warrants further research.

These findings align with a broader Palearctic trend, where taxa historically classified as single, polytypic species are increasingly revealed to be complexes of deeply diverged lineages. This pattern is well-documented in species like *Montifringilla nivalis* (Islam et al., 2024), *Troglodytes troglodytes* (Albrecht et al., 2020), and *Sitta europaea* (Päckert et al., 2020). Within the Corvidae, such deep east–west divergences (Haring, Gamauf & Kryukov, 2007) are further corroborated by complex phylogeographic structures in *Pica pica* (Song et al., 2018; Kryukov et al., 2022), *Garrulus glandarius* (Wolf et al., 2026), and *Nucifraga caryocatactes* (de Raad et al., 2022), placing the divergence of chough lineages into a robust regional context.

### Study limitations

Although this study represents the most geographically comprehensive mitochondrial sampling conducted to date for both *Pyrrhocorax* species, covering nearly all peripheral parts of their ranges and their most isolated populations, important geographic gaps remain that could further refine our understanding of their biogeographic histories. The lack of genetic data from key regions for *P. graculus*, including the Middle Eastern cluster such as Anatolia, the Caucasus and the Iranian highlands, the easternmost margin of the Tibetan Plateau, and major Central Asian mountain systems including the Tian Shan, Pamir and Altai ranges, limits our ability to determine whether these areas contributed to diversification events or whether individuals from these regions belong to any of the genetic clusters identified in this study. For *P. pyrrhocorax*, genetic information is also missing from several critical areas, including the easternmost parts of its range in China, the Afghan and Central Asian Mountain ranges, the Caucasian–Zagros region, the northern limit of its distribution in the Sayan Mountains (southern Siberia), and the Balkans. Thus, diversity indices are conditioned by sampling bias; while certain regions are spatially well-represented, others suffer from undersampling that can skew local diversity estimates.

Herein molecular clock divergence estimates are in good accordance with that inferred from independent-rate-based time calibration by Fernando, Peterson & Li (2017), thus divergence times estimated from fossil calibrations obtained much preterit estimates (Jønsson et al., 2016; Zhang et al., 2026) In addition, molecular clock estimates are inherently uncertain due to limitations in fossil calibration and assumptions about empirical mutation rates can substantially underestimate divergence times (Ho et al., 2005; Warnock, Yang & Donoghue, 2012; Zhu, Dos Rei & Yang, 2015). Fossil records of choughs, including Lower Pliocene remains from Hungary dated to around 3.7 Mya (Kessler, 2013; Kessler, 2020), Upper Pliocene remains from Bulgaria at approximately 2.6 Mya (Boev, 2000), and MIS 22 fossils from the Iberian Peninsula, roughly 890 kya (Sánchez-Marco, 2012), predate the inferred arrival of the genus in Europe. This discrepancy may reflect limitations in fossil identification and dating, in molecular clock calibration, or a more complex paleobiogeographic history involving multiple dispersal events across Eurasia followed by local extinctions. Future ancient DNA analyses may clarify whether these fossils represent extant lineages or now extinct evolutionary branches (Billerman & Walsh, 2019).

## Conclusions

The results of this study provide a detailed phylogeographic framework for *Pyrrhocorax* choughs, highlighting highly structured mitochondrial lineages shaped by historical isolation, environmental change, and ecological constraints. Quantitative assessments of diversity and divergence reinforce this architecture, showing that dispersal capacity and ecological plasticity govern population connectivity. However, while mitochondrial markers reveal deep phylogeographic splits, BSD models indicates these structured lineages do not fully reflect independent lineage boundaries, pointing instead to recent or ongoing gene flow. The Mediterranean basin and North Africa served as vital climatic refugia for choughs during Pleistocene glaciations; these regions facilitated population persistence amidst habitat fragmentation, making them cornerstone areas for the long-term conservation of the species’ evolutionary heritage. Despite their historical significance, however, the current population status and demographic trends remain virtually undocumented across North Africa and the Middle East. Within Europe, where population trends are better known, populations of *P. pyrrhocorax* from the Iberian Peninsula, the Alps, and other western mountain, coastal, and insular systems, including La Palma and the British Isles, are divided into three distinct mitochondrial lineages, whereas populations from southeastern Europe and the Middle East, including Greece, Turkey, and Iran, form a single, distinct lineage. Conservation efforts for extinct, declining, or fragmented populations should prioritize their respective lineage in captive breeding, reintroduction, or reinforcement programs to preserve local adaptation, avoid genetic homogenization, and maximize long-term population viability. Other aspects of local adaptation reflected in the nuclear genome are more complex to account for due to the species’ marked genetic structuring driven by social isolation processes (Morinha et al., 2017), which can lead to rapid selection, genetic drift, and inbreeding with potential consequences for population health when numbers are greatly reduced (Pennycott, Bignal & McCracken, 2025). This information, combined with morphological and ecological data, will help clarify in future studies the taxonomic status of these species at the subspecies level, which are commonly used as conservation units in management and conservation planning.

## Supplemental Information

10.7717/peerj.21799/supp-1 Supplemental Information 1 Specimen identities sequenced in this study Voucher reference numbers, species identity, sampling localities, and GenBank accession numbers of the control region (*CR*), the NADH dehydrogenase subunit 2 (*ND2*), and the cytochrome c oxidase subunit I (*COI*), and the transforming growth factor β 2 (*TGFB2*), and the Brahma gene (*BRM*).

10.7717/peerj.21799/supp-2 Supplemental Information 2 Maximum likelihood marginal probabilities (%) of each ancestral geographic state at the bottom of the branches below each node (Nodes 3–13), inferred using the *BioGeoBEARS* package Geographic areas are coded as follows: (A) eastern Europe and the Middle East; (B) Ethiopia; (C) western Europe (Mediterranean and UK); (D) La Palma, Canary Islands; (E) Kazakhstan and Mongolia (Eastern Asia); (F) Himalayas; (G) Morocco (northwestern Africa). Combinations represent ancestral widespread/multiregional ranges. Node codes are referred to Table 3.

## Additional Information and Declarations

### Field Study Permissions

The following information was supplied relating to field study approvals (i.e., approving body and any reference numbers): Blood samples from Spain were obtained under official permits from the Spanish Ministry of the Environment and Regional Administrations, linked to capture, handling, and ringing permits from the Spanish Bird Ringing Centre (permit no. 530115 to G.B.).

### Data Availability

The following information was supplied regarding data availability: The data is available in the Supplemental Files.
