# Supplementary material for: Comparative range-wide phylogeography and evolutionary history of the two Pyrrhocorax species (Passeriformes: Corvidae)
Source: PeerJ. 2026 Oct 9;14:e21799. doi: 10.7717/peerj.21799 (PMC13657079; doi:10.7717/peerj.21799)
Supplement: Supplementary file 1 [file peerj-14-21799-s001.docx]

**Table S1.** Specimen identities sequenced in this study. Voucher reference numbers, species identity, sampling localities, and GenBank accession numbers of the control region (*CR*), the NADH dehydrogenase subunit 2 (*ND2*), and the cytochrome c oxidase subunit I (*COI*), and the transforming growth factor β 2 (*TGFB2*), and the Brahma gene (*BRM*).

| **Voucher** | **Species** | **Sampling location** | ***COI*** | ***ND2*** | ***CR*** | ***TGFB2*** | ***BRM*** | **Type** |
| --- | --- | --- | --- | --- | --- | --- | --- | --- |
| PpyHIM1 | *P. pyrrhocorax* | Manang, Nepal | PZ092200 | PZ095632 | PZ095730 | PZ101992 | PZ102089 | Feather |
| PpyHIM4 | *P. pyrrhocorax* | Manang, Nepal | PZ092201 | PZ095633 | PZ095731 | PZ101993 | PZ102090 | Feather |
| PpyMONG1 | *P. pyrrhocorax* | Altai Tavan Bogd National Park, Mongolia | PZ092182 | PZ095614 | PZ095712 | PZ101974 | PZ102071 | Feather |
| PpyMONG2 | *P. pyrrhocorax* | Altai Tavan Bogd National Park, Mongolia | PZ092183 | PZ095615 | PZ095713 | PZ101975 | PZ102072 | Feather |
| PpyMONG3 | *P. pyrrhocorax* | Onon-Balzj National Park, Mongolia | PZ092184 | PZ095616 | PZ095714 | PZ101976 | PZ102073 | Feather |
| PpyMONG4 | *P. pyrrhocorax* | Onon-Balzj National Park, Mongolia | PZ092185 | PZ095617 | PZ095715 | PZ101977 | PZ102074 | Feather |
| PpyMONG5 | *P. pyrrhocorax* | Onon-Balzj National Park, Mongolia | PZ092186 | PZ095618 | PZ095716 | PZ101978 | PZ102075 | Feather |
| PpyMONG6 | *P. pyrrhocorax* | Tarvagatai Mountains, Mongolia | PZ092187 | PZ095619 | PZ095717 | PZ101979 | PZ102076 | Feather |
| PpyMONG7 | *P. pyrrhocorax* | Tarvagatai Mountains, Mongolia | PZ092188 | PZ095620 | PZ095718 | PZ101980 | PZ102077 | Feather |
| PpyMONG8 | *P. pyrrhocorax* | Tarvagatai Mountains, Mongolia | PZ092189 | PZ095621 | PZ095719 | PZ101981 | PZ102078 | Feather |
| PpyMONG9 | *P. pyrrhocorax* | Terejil National Park, Mongolia | PZ092190 | PZ095622 | PZ095720 | PZ101982 | PZ102079 | Feather |
| PpyMONG10 | *P. pyrrhocorax* | Terejil National Park, Mongolia | PZ092191 | PZ095623 | PZ095721 | PZ101983 | PZ102080 | Feather |
| PpyMONG11 | *P. pyrrhocorax* | Terejil National Park, Mongolia | PZ092192 | PZ095624 | PZ095722 | PZ101984 | PZ102081 | Feather |
| PpyKAZ1 | *P. pyrrhocorax* | Kazakhsthan | PZ092181 | PZ095613 | PZ095711 | PZ101973 | PZ102070 | Blood |
| PpyKAZ5 | *P. pyrrhocorax* | Kazakhsthan | PZ092145 | PZ095577 | PZ095675 | PZ101940 | PZ102034 | Blood |
| PpyIRAN1 | *P. pyrrhocorax* | Sarakhs, NE Iran | PZ092166 | PZ095598 | PZ095696 | PZ101959 | PZ102055 | Feather |
| PpyIRAN2 | *P. pyrrhocorax* | Sabzevar, NE Iran | PZ092180 | PZ095612 | PZ095710 | PZ101972 | PZ102069 | Feather |
| PpyIRAN3 | *P. pyrrhocorax* | Kashmar, E Iran | PZ092167 | PZ095599 | PZ095697 | PZ101960 | PZ102056 | Feather |
| PpyIRAN4 | *P. pyrrhocorax* | Helali Protected Area, E Iran | PZ092168 | PZ095600 | PZ095698 | PZ101961 | PZ102057 | Feather |
| PpyIRAN6 | *P. pyrrhocorax* | Maleh-Galeh Protected Area, SW Iran | PZ092169 | PZ095601 | PZ095699 | PZ101962 | PZ102058 | Feather |
| PpyTK1 | *P. pyrrhocorax* | Taurus Mountains, Turkey | PZ092178 | PZ095610 | PZ095708 | PZ101971 | PZ102067 | Feather |
| PpyTK2 | *P. pyrrhocorax* | Taurus Mountains, Turkey | PZ092179 | PZ095611 | PZ095709 | NA | PZ102068 | Feather |
| PpyGR14 | *P. pyrrhocorax* | Crete, Greece | PZ092198 | PZ095630 | PZ095728 | PZ101990 | PZ102087 | Feather |
| PpyGR3 | *P. pyrrhocorax* | Crete, Greece | PZ092197 | PZ095629 | PZ095727 | PZ101989 | PZ102086 | Feather |
| PpyGR19 | *P. pyrrhocorax* | Crete, Greece | PZ092199 | PZ095631 | PZ095729 | PZ101991 | PZ102088 | Feather |
| PpyET1 | *P. pyrrhocorax* | Bale Mountains, Ethiopia | PZ092193 | PZ095625 | PZ095723 | PZ101985 | PZ102082 | Feather |
| PpyET2 | *P. pyrrhocorax* | Bale Mountains, Ethiopia | PZ092194 | PZ095626 | PZ095724 | PZ101986 | PZ102083 | Feather |
| PpyET3 | *P. pyrrhocorax* | Bale Mountains, Ethiopia | PZ092195 | PZ095627 | PZ095725 | PZ101987 | PZ102084 | Feather |
| PpyET5 | *P. pyrrhocorax* | Bale Mountains, Ethiopia | PZ092196 | PZ095628 | PZ095726 | PZ101988 | PZ102085 | Feather |
| PpyMOR2 | *P. pyrrhocorax* | Tizi n’ Tichka, Haouz, Marrakech-Tensift-El Haouz, Morocco | PZ092146 | PZ095578 | PZ095676 | PZ101941 | PZ102035 | Feather |
| PpyMOR3 | *P. pyrrhocorax* | Tizi n’ Tichka, Haouz, Marrakech-Tensift-El Haouz, Morocco | PZ092147 | PZ095579 | PZ095677 | PZ101942 | PZ102036 | Feather |
| PpyCANT1 | *P. pyrrhocorax* | El-Juein, Souk, Tleta, Tagmramet, Morocco | PZ092150 | PZ095582 | PZ095680 | NA | PZ102039 | Feather |
| PpyCANT2 | *P. pyrrhocorax* | El-Juein, Souk, Tleta, Tagmramet, Morocco | PZ092151 | PZ095583 | PZ095681 | NA | PZ102040 | Feather |
| PpyCANT17 | *P. pyrrhocorax* | El-Juein, Souk, Tleta, Tagmramet, Morocco | PZ092152 | PZ095584 | PZ095682 | PZ101945 | PZ102041 | Feather |
| PpyCANT18 | *P. pyrrhocorax* | Cantera El-Juein, Souk, Tleta, Tagmramet, Morocco | PZ092153 | PZ095585 | PZ095683 | PZ101946 | PZ102042 | Feather |
| PpyAE1 | *P. pyrrhocorax* | Andradia, Elgarra, Morocco | PZ092148 | PZ095580 | PZ095678 | PZ101943 | PZ102037 | Feather |
| PpyAE2 | *P. pyrrhocorax* | Andradia, Elgarra, Morocco | PZ092149 | PZ095581 | PZ095679 | PZ101944 | PZ102038 | Feather |
| PpyLP006 | *P. pyrrhocorax* | La Palma, Canary Islands | PZ092137 | PZ095569 | PZ095667 | PZ101932 | PZ102026 | Blood |
| PpyLP011 | *P. pyrrhocorax* | La Palma, Canary Islands | PZ092138 | PZ095570 | PZ095668 | PZ101933 | PZ102027 | Blood |
| PpyLP031 | *P. pyrrhocorax* | La Palma, Canary Islands | PZ092142 | PZ095574 | PZ095672 | PZ101937 | PZ102031 | Blood |
| PpyLP012 | *P. pyrrhocorax* | La Palma, Canary Islands | PZ092177 | PZ095609 | PZ095707 | PZ101970 | PZ102066 | Blood |
| PpyLP019 | *P. pyrrhocorax* | La Palma, Canary Islands | PZ092139 | PZ095571 | PZ095669 | PZ101934 | PZ102028 | Blood |
| PpyLP024 | *P. pyrrhocorax* | La Palma, Canary Islands | PZ092140 | PZ095572 | PZ095670 | PZ101935 | PZ102029 | Blood |
| PpyLP029 | *P. pyrrhocorax* | La Palma, Canary Islands | PZ092141 | PZ095573 | PZ095671 | PZ101936 | PZ102030 | Blood |
| PpyLP057 | *P. pyrrhocorax* | La Palma, Canary Islands | PZ092143 | PZ095575 | PZ095673 | PZ101938 | PZ102032 | Blood |
| PpyLP062 | *P. pyrrhocorax* | La Palma, Canary Islands | PZ092144 | PZ095576 | PZ095674 | PZ101939 | PZ102033 | Blood |
| PpyUK1 | *P. pyrrhocorax* | Fort Island, Isle of Man, United Kingdom | PZ092161 | PZ095593 | PZ095691 | PZ101954 | PZ102050 | Feather |
| PpyUK3 | *P. pyrrhocorax* | Contrary Head, Peel Hill, Isle of Man, United Kingdom | PZ092162 | PZ095594 | PZ095692 | PZ101955 | PZ102051 | Feather |
| PpyUK5 | *P. pyrrhocorax* | Shennvalley, Isle of Man, United Kingdom | PZ092163 | PZ095595 | PZ095693 | PZ101956 | PZ102052 | Feather |
| PpyUK6 | *P. pyrrhocorax* | Claghbane, Isle of Man, United Kingdom | PZ092164 | PZ095596 | PZ095694 | PZ101957 | PZ102053 | Feather |
| PpyUK8 | *P. pyrrhocorax* | Aldrick, Isle of Man, United Kingdom | PZ092165 | PZ095597 | PZ095695 | PZ101958 | PZ102054 | Feather |
| PpyIT1 | *P. pyrrhocorax* | Val d'Aosta, Italy | PZ092160 | PZ095592 | PZ095690 | PZ101953 | PZ102049 | Blood |
| PpyIT4 | *P. pyrrhocorax* | Gole del Sagitario, Italy | PZ092154 | PZ095586 | PZ095684 | PZ101947 | PZ102043 | Feather |
| PpyIT6 | *P. pyrrhocorax* | Gole del Sagitario, Italy | PZ092155 | PZ095587 | PZ095685 | PZ101948 | PZ102044 | Feather |
| PpyIT8 | *P. pyrrhocorax* | Gole del Sagitario, Italy | PZ092156 | PZ095588 | PZ095686 | PZ101949 | PZ102045 | Feather |
| PpyIT9 | *P. pyrrhocorax* | Gole del Sagitario, Italy | PZ092157 | PZ095589 | PZ095687 | PZ101950 | PZ102046 | Feather |
| PpyIT10 | *P. pyrrhocorax* | Gole del Sagitario, Italy | PZ092158 | PZ095590 | PZ095688 | PZ101951 | PZ102047 | Feather |
| PpyIT11 | *P. pyrrhocorax* | Santa Maria, Gole di Celano, Italy | PZ092159 | PZ095591 | PZ095689 | PZ101952 | PZ102048 | Feather |
| PpyAWC | *P. pyrrhocorax* | La Aldehuela, Madrid, Spain | PZ092174 | PZ095606 | PZ095704 | PZ101967 | PZ102063 | Blood |
| PpyAUW | *P. pyrrhocorax* | La Aldehuela, Madrid, Spain | PZ092172 | PZ095604 | PZ095702 | PZ101965 | PZ102061 | Blood |
| PpyAVT | *P. pyrrhocorax* | La Aldehuela, Madrid, Spain | PZ092173 | PZ095605 | PZ095703 | PZ101966 | PZ102062 | Blood |
| Ppy607 | *P. pyrrhocorax* | Los Monegros, Zaragoza, Spain | PZ092170 | PZ095602 | PZ095700 | PZ101963 | PZ102059 | Blood |
| Ppy1TL | *P. pyrrhocorax* | Los Monegros, Zaragoza, Spain | PZ092171 | PZ095603 | PZ095701 | PZ101964 | PZ102060 | Blood |
| PpyMTJ32 | *P. pyrrhocorax* | Montejaque, Malaga, Spain | PZ092176 | PZ095608 | PZ095706 | PZ101969 | PZ102065 | Feather |
| PpyMTJ5 | *P. pyrrhocorax* | Montejaque, Malaga, Spain | PZ092175 | PZ095607 | PZ095705 | PZ101968 | PZ102064 | Feather |
| PgrIND | *P. graculus* | Leh, India | PZ092227 | PZ095659 | PZ095757 | PZ102018 | PZ102116 | Feather |
| PgrAtlas | *P. graculus* | Atlas, Morocco | PZ092207 | PZ095639 | PZ095737 | PZ101998 | PZ102096 | Feather |
| PgrT1 | *P. graculus* | Taghramt, Morocco | PZ092221 | PZ095653 | PZ095751 | PZ102012 | PZ102110 | Feather |
| PgrT2 | *P. graculus* | Taghramt, Morocco | PZ092208 | PZ095640 | PZ095738 | PZ101999 | PZ102097 | Feather |
| PgrDAL1 | *P. graculus* | Dalgasi, Morocco | PZ092222 | PZ095654 | PZ095752 | PZ102013 | PZ102111 | Feather |
| PgrDAL2 | *P. graculus* | Dalgasi, Morocco | PZ092209 | PZ095641 | PZ095739 | PZ102000 | PZ102098 | Feather |
| PgrSTT3 | *P. graculus* | Souk, Tleta, Taghuame, Morocco | PZ092211 | PZ095643 | PZ095741 | PZ102002 | PZ102100 | Feather |
| PgrCANT6 | *P. graculus* | El-Juein, Souk, Tleta, Tagmramet, Morocco | PZ092210 | PZ095642 | PZ095740 | PZ102001 | PZ102099 | Feather |
| Pgr5120558 | *P. graculus* | Picos de Europa, Asturias, Spain | PZ092213 | PZ095645 | PZ095743 | PZ102004 | PZ102102 | Feather |
| Pgr5120553 | *P. graculus* | Picos de Europa, Asturias, Spain | PZ092224 | PZ095656 | PZ095754 | PZ102015 | PZ102113 | Feather |
| Pgr5120554 | *P. graculus* | Picos de Europa, Asturias, Spain | PZ092225 | PZ095657 | PZ095755 | PZ102016 | PZ102114 | Feather |
| Pgr5110215 | *P. graculus* | Picos de Europa, Asturias, Spain | PZ092212 | PZ095644 | PZ095742 | PZ102003 | PZ102101 | Feather |
| PgrAL37 | *P. graculus* | Picos de Europa, Asturias, Spain | PZ092214 | PZ095646 | PZ095744 | PZ102005 | PZ102103 | Feather |
| PgrVA14 | *P. graculus* | Picos de Europa, Asturias, Spain | PZ092215 | PZ095647 | PZ095745 | PZ102006 | PZ102104 | Feather |
| PgrPIR1 | *P. graculus* | Pirineos, Huesca, Spain | PZ092233 | PZ095665 | PZ095763 | PZ102024 | PZ102122 | Feather |
| PgrPIR2 | *P. graculus* | Pirineos, Huesca, Spain | PZ092234 | PZ095666 | PZ095764 | PZ102025 | PZ102123 | Feather |
| PgrBO | *P. graculus* | Gran Paradiso, Val Montey, Italy | PZ092202 | PZ095634 | PZ095732 | PZ101994 | PZ102091 | Blood |
| PgrBA | *P. graculus* | Gran Paradiso, Val Montey, Italy | PZ092203 | PZ095635 | PZ095733 | PZ101995 | PZ102092 | Blood |
| PgrVV | *P. graculus* | Gran Paradiso, Val Montey, Italy | PZ092223 | PZ095655 | PZ095753 | PZ102014 | PZ102112 | Blood |
| PgrAU1 | *P. graculus* | Kaiser-Franz-Josefs-Höhe, Austria | PZ092216 | PZ095648 | PZ095746 | PZ102007 | PZ102105 | Feather |
| PgrAU2 | *P. graculus* | Kaiser-Franz-Josefs-Höhe, Austria | PZ092217 | PZ095649 | PZ095747 | PZ102008 | PZ102106 | Feather |
| PgrAU3 | *P. graculus* | Kaiser-Franz-Josefs-Höhe, Austria | PZ092218 | PZ095650 | PZ095748 | PZ102009 | PZ102107 | Feather |
| PgrAU4 | *P. graculus* | Kaiser-Franz-Josefs-Höhe, Austria | PZ092219 | PZ095651 | PZ095749 | PZ102010 | PZ102108 | Feather |
| PgrAU5 | *P. graculus* | Kaiser-Franz-Josefs-Höhe, Austria | PZ092226 | PZ095658 | PZ095756 | PZ102017 | PZ102115 | Feather |
| PgrAU6 | *P. graculus* | Kaiser-Franz-Josefs-Höhe, Austria | PZ092220 | PZ095652 | PZ095750 | PZ102011 | PZ102109 | Feather |
| PgrGR1 | *P. graculus* | Pindos Mountains, Greece | PZ092228 | PZ095660 | PZ095758 | PZ102019 | PZ102117 | Feather |
| PgrGR5 | *P. graculus* | Crete, Greece | PZ092229 | PZ095661 | PZ095759 | PZ102020 | PZ102118 | Feather |
| PgrGR6 | *P. graculus* | Crete, Greece | PZ092230 | PZ095662 | PZ095760 | PZ102021 | PZ102119 | Feather |
| PgrGR11 | *P. graculus* | Crete, Greece | PZ092231 | PZ095663 | PZ095761 | PZ102022 | PZ102120 | Feather |
| PgrGR13 | *P. graculus* | Crete, Greece | PZ092232 | PZ095664 | PZ095762 | PZ102023 | PZ102121 | Feather |
| PgrBUL1 | *P. graculus* | The Central Balkan National Park, Bulgaria | PZ092204 | PZ095636 | PZ095734 | NA | PZ102093 | Feather |
| PgrBUL5 | *P. graculus* | The Central Balkan National Park, Bulgaria | PZ092205 | PZ095637 | PZ095735 | PZ101996 | PZ102094 | Feather |
| PgrBUL6 | *P. graculus* | The Central Balkan National Park, Bulgaria | PZ092206 | PZ095638 | PZ095736 | PZ101997 | PZ102095 | Feather |
