# Supplementary material for: Comparative range-wide phylogeography and evolutionary history of the two Pyrrhocorax species (Passeriformes: Corvidae)
Source: PeerJ. 2026 Oct 9;14:e21799. doi: 10.7717/peerj.21799 (PMC13657079; doi:10.7717/peerj.21799)
Supplement: Supplementary file 2 [file peerj-14-21799-s002.docx]

**Table S2.** Maximum likelihood marginal probabilities (%) of each ancestral geographic state at the bottom of the branches below each node (Nodes 3–13), inferred using the *BioGeoBEARS* package. Geographic areas are coded as follows: (A) eastern Europe and the Middle East; (B) Ethiopia; (C) western Europe (Mediterranean and UK); (D) La Palma, Canary Islands; (E) Kazakhstan and Mongolia (Eastern Asia); (F) Himalayas; (G) Morocco (northwestern Africa). Combinations represent ancestral widespread/multiregional ranges. Node codes are referred to Table 3.

| **Geographic area** | **Node 3** | **Node 4** | **Node 5** | **Node 6** | **Node 7** | **Node 8** | **Node 9** | **Node 10** | **Node 11** | **Node 12** | **Node 13** |
| --- | --- | --- | --- | --- | --- | --- | --- | --- | --- | --- | --- |
| - | 0.000 | 0.000 | 0.000 | 0.000 | 0.000 | 0.000 | 0.000 | 0.000 | 0.000 | 0.000 | 0.000 |
| A | 11.801 | 11.110 | 2.105 | 15.625 | 0.452 | 0.235 | 12.632 | 0.442 | 1.157 | 1.074 | 0.000 |
| B | 3.415 | 6.240 | 1.696 | 10.498 | 4.593 | 1.035 | 0.739 | 0.870 | 0.978 | 0.000 | 0.000 |
| C | 9.907 | 7.151 | 1.399 | 7.625 | 2.957 | 14.640 | 11.948 | 61.847 | 0.875 | 1.013 | 99.999 |
| D | 2.707 | 3.036 | 0.793 | 2.373 | 0.841 | 3.707 | 0.690 | 12.732 | 0.624 | 0.000 | 0.000 |
| E | 3.962 | 8.713 | 26.617 | 0.690 | 0.033 | 0.066 | 0.776 | 0.074 | 68.308 | 0.000 | 0.000 |
| F | 16.898 | 14.307 | 28.006 | 0.734 | 0.034 | 0.068 | 3.586 | 0.077 | 14.551 | 0.000 | 0.000 |
| G | 10.272 | 7.770 | 1.516 | 8.737 | 3.369 | 16.409 | 12.225 | 4.137 | 0.921 | 0.002 | 0.000 |
| AB | 0.772 | 1.364 | 0.027 | 10.287 | 0.609 | 0.028 | 0.826 | 0.018 | 0.038 | 0.018 | 0.000 |
| AC | 2.010 | 1.211 | 0.028 | 7.416 | 0.436 | 1.090 | 10.637 | 0.622 | 0.036 | 96.015 | 0.000 |
| AD | 0.729 | 0.751 | 0.025 | 2.330 | 0.120 | 0.286 | 0.780 | 0.200 | 0.029 | 0.018 | 0.000 |
| AE | 0.805 | 1.989 | 0.794 | 0.394 | 0.005 | 0.005 | 0.862 | 0.001 | 1.428 | 0.019 | 0.000 |
| AF | 3.020 | 2.220 | 0.841 | 0.421 | 0.005 | 0.006 | 1.354 | 0.001 | 0.174 | 0.037 | 0.000 |
| AG | 2.040 | 1.295 | 0.029 | 8.486 | 0.488 | 1.178 | 10.821 | 0.031 | 0.037 | 0.039 | 0.000 |
| BC | 0.736 | 0.888 | 0.025 | 4.399 | 23.618 | 1.719 | 0.787 | 1.751 | 0.030 | 0.015 | 0.000 |
| BD | 0.258 | 0.552 | 0.022 | 1.328 | 6.786 | 0.450 | 0.057 | 0.561 | 0.024 | 0.000 | 0.000 |
| BE | 0.285 | 1.489 | 0.702 | 0.251 | 0.248 | 0.009 | 0.063 | 0.003 | 1.157 | 0.000 | 0.000 |
| BF | 1.076 | 1.689 | 0.750 | 0.270 | 0.259 | 0.009 | 0.099 | 0.003 | 0.150 | 0.000 | 0.000 |
| BG | 0.748 | 0.947 | 0.026 | 5.008 | 27.005 | 1.857 | 0.811 | 0.057 | 0.031 | 0.000 | 0.000 |
| CD | 0.693 | 0.468 | 0.023 | 0.411 | 1.381 | 3.380 | 0.740 | 12.203 | 0.021 | 0.015 | 0.000 |
| CE | 0.769 | 1.326 | 0.725 | 0.183 | 0.177 | 0.134 | 0.822 | 0.108 | 1.029 | 0.015 | 0.000 |
| CF | 2.883 | 1.481 | 0.767 | 0.196 | 0.185 | 0.139 | 1.285 | 0.110 | 0.139 | 0.031 | 0.000 |
| CG | 1.942 | 0.810 | 0.026 | 2.882 | 13.456 | 38.732 | 10.299 | 2.492 | 0.029 | 0.032 | 0.001 |
| DE | 0.270 | 0.791 | 0.622 | 0.057 | 0.049 | 0.035 | 0.060 | 0.035 | 0.723 | 0.000 | 0.000 |
| DF | 1.017 | 0.899 | 0.665 | 0.061 | 0.051 | 0.037 | 0.093 | 0.036 | 0.107 | 0.000 | 0.000 |
| DG | 0.705 | 0.519 | 0.023 | 0.870 | 3.757 | 10.448 | 0.764 | 0.796 | 0.023 | 0.000 | 0.000 |
| EF | 1.121 | 2.217 | 23.419 | 0.011 | 0.002 | 0.001 | 0.103 | 0.000 | 5.129 | 0.000 | 0.000 |
| EG | 0.781 | 1.425 | 0.735 | 0.209 | 0.198 | 0.145 | 0.847 | 0.005 | 1.087 | 0.000 | 0.000 |
| FG | 2.926 | 1.591 | 0.778 | 0.223 | 0.207 | 0.150 | 1.325 | 0.005 | 0.145 | 0.000 | 0.000 |
| ABC | 0.317 | 0.351 | 0.003 | 0.952 | 1.620 | 0.062 | 0.226 | 0.001 | 0.000 | 0.001 | 0.000 |
| ABD | 0.109 | 0.175 | 0.002 | 0.280 | 0.426 | 0.016 | 0.016 | 0.000 | 0.000 | 0.000 | 0.000 |
| ABE | 0.145 | 0.561 | 0.073 | 0.555 | 0.015 | 0.000 | 0.017 | 0.000 | 0.008 | 0.000 | 0.000 |
| ABF | 0.610 | 0.875 | 0.074 | 0.589 | 0.016 | 0.000 | 0.147 | 0.000 | 0.005 | 0.000 | 0.000 |
| ABG | 0.327 | 0.374 | 0.003 | 1.082 | 1.779 | 0.057 | 0.236 | 0.001 | 0.000 | 0.000 | 0.000 |
| ACD | 0.263 | 0.192 | 0.002 | 0.079 | 0.083 | 0.010 | 0.213 | 0.012 | 0.000 | 0.001 | 0.000 |
| ACE | 0.359 | 0.630 | 0.069 | 0.401 | 0.013 | 0.001 | 0.237 | 0.000 | 0.008 | 0.001 | 0.000 |
| ACF | 1.773 | 0.870 | 0.069 | 0.421 | 0.014 | 0.001 | 2.009 | 0.000 | 0.004 | 0.001 | 0.000 |
| ACG | 0.881 | 0.378 | 0.003 | 0.582 | 0.906 | 0.154 | 4.366 | 0.024 | 0.000 | 1.652 | 0.000 |
| ADE | 0.125 | 0.291 | 0.050 | 0.123 | 0.003 | 0.000 | 0.016 | 0.000 | 0.006 | 0.000 | 0.000 |
| ADF | 0.522 | 0.456 | 0.050 | 0.130 | 0.004 | 0.000 | 0.139 | 0.000 | 0.003 | 0.000 | 0.000 |
| ADG | 0.272 | 0.211 | 0.002 | 0.173 | 0.245 | 0.039 | 0.221 | 0.006 | 0.000 | 0.000 | 0.000 |
| AEF | 0.677 | 1.253 | 1.959 | 0.034 | 0.000 | 0.000 | 0.154 | 0.000 | 0.244 | 0.000 | 0.000 |
| AEG | 0.369 | 0.679 | 0.073 | 0.457 | 0.014 | 0.001 | 0.247 | 0.000 | 0.008 | 0.000 | 0.000 |
| AFG | 1.825 | 0.937 | 0.072 | 0.479 | 0.015 | 0.001 | 2.090 | 0.000 | 0.004 | 0.001 | 0.000 |
| BCD | 0.091 | 0.107 | 0.002 | 0.024 | 0.215 | 0.150 | 0.015 | 0.035 | 0.000 | 0.000 | 0.000 |
| BCE | 0.128 | 0.350 | 0.052 | 0.204 | 0.020 | 0.007 | 0.017 | 0.000 | 0.007 | 0.000 | 0.000 |
| BCF | 0.535 | 0.547 | 0.052 | 0.217 | 0.020 | 0.007 | 0.140 | 0.000 | 0.004 | 0.000 | 0.000 |
| BCG | 0.280 | 0.222 | 0.002 | 0.125 | 2.474 | 2.209 | 0.223 | 0.070 | 0.000 | 0.000 | 0.000 |
| BDE | 0.040 | 0.135 | 0.035 | 0.062 | 0.005 | 0.002 | 0.001 | 0.000 | 0.005 | 0.000 | 0.000 |
| BDF | 0.154 | 0.273 | 0.035 | 0.066 | 0.005 | 0.002 | 0.010 | 0.000 | 0.003 | 0.000 | 0.000 |
| BDG | 0.095 | 0.116 | 0.002 | 0.042 | 0.660 | 0.565 | 0.015 | 0.016 | 0.000 | 0.000 | 0.000 |
| BEF | 0.203 | 0.756 | 1.331 | 0.021 | 0.000 | 0.000 | 0.011 | 0.000 | 0.196 | 0.000 | 0.000 |
| BEG | 0.132 | 0.376 | 0.055 | 0.232 | 0.021 | 0.007 | 0.017 | 0.000 | 0.007 | 0.000 | 0.000 |
| BFG | 0.553 | 0.587 | 0.055 | 0.246 | 0.022 | 0.007 | 0.146 | 0.000 | 0.004 | 0.000 | 0.000 |
| CDE | 0.107 | 0.173 | 0.028 | 0.020 | 0.001 | 0.001 | 0.016 | 0.002 | 0.005 | 0.000 | 0.000 |
| CDF | 0.448 | 0.272 | 0.028 | 0.021 | 0.001 | 0.001 | 0.132 | 0.002 | 0.003 | 0.000 | 0.000 |
| CDG | 0.226 | 0.125 | 0.002 | 0.013 | 0.050 | 0.659 | 0.209 | 0.606 | 0.000 | 0.000 | 0.000 |
| CEF | 0.603 | 0.791 | 0.992 | 0.015 | 0.000 | 0.000 | 0.146 | 0.000 | 0.173 | 0.000 | 0.000 |
| CEG | 0.323 | 0.404 | 0.049 | 0.134 | 0.011 | 0.018 | 0.234 | 0.004 | 0.007 | 0.000 | 0.000 |
| CFG | 1.593 | 0.559 | 0.049 | 0.140 | 0.011 | 0.018 | 1.986 | 0.004 | 0.003 | 0.000 | 0.000 |
| DEF | 0.175 | 0.367 | 0.350 | 0.005 | 0.000 | 0.000 | 0.010 | 0.000 | 0.119 | 0.000 | 0.000 |
| DEG | 0.111 | 0.195 | 0.031 | 0.041 | 0.003 | 0.004 | 0.016 | 0.001 | 0.005 | 0.000 | 0.000 |
| DFG | 0.464 | 0.306 | 0.031 | 0.043 | 0.003 | 0.005 | 0.137 | 0.001 | 0.003 | 0.000 | 0.000 |
| EFG | 0.621 | 0.858 | 1.126 | 0.017 | 0.000 | 0.000 | 0.153 | 0.000 | 0.183 | 0.000 | 0.000 |
